# Covariation MS uncovers a protein that controls cysteine catabolism

**DOI:** 10.1038/s41586-025-09535-5

**Published:** 2025-09-17

**Authors:** Haopeng Xiao, Martha Ordonez, Emma C. Fink, Taylor A. Covington, Hilina B. Woldemichael, Junyi Chen, Mika Sarkin Jain, Milan H. Rohatgi, Shelley M. Wei, Nils Burger, Muneeb A. Sharif, Julius Jan, Yaoyu Wang, Jonathan J. Petrocelli, Katherine Blackmore, Amanda L. Smythers, Bingsen Zhang, Matthew Gilbert, Hakyung Cheong, Sumeet A. Khetarpal, Arianne Smith, Dina Bogoslavski, Yu Lei, Laura Pontano Vaites, Fiona E. McAllister, Nick Van Bruggen, Katherine A. Donovan, Edward L. Huttlin, Evanna L. Mills, Eric S. Fischer, Edward T. Chouchani

**Affiliations:** 1 Department of Cancer Biology, Dana–Farber Cancer Institute, Boston, MA USA; 2 Department of Cell Biology, Harvard Medical School, Boston, MA USA; 3 Department of Biochemistry, Stanford University School of Medicine, Stanford, CA USA; 4 Stanford Cancer Institute, Stanford University School of Medicine, Stanford, CA USA; 5 Department of Medical Oncology, Dana–Farber Cancer Institute, Boston, MA USA; 6 Department of Biological Chemistry and Molecular Pharmacology, Harvard Medical School, Boston, MA USA; 7 Department of Computer Science, Stanford University, Stanford, CA USA; 8 Harvard Medical School, Boston, MA USA; 9 Calico Life Sciences, South San Francisco, CA USA; 10 Department of Cancer Immunology and Virology, Dana–Farber Cancer Institute, Boston, MA USA; 11 Department of Immunology, Harvard Medical School, Boston, MA USA; 12 Howard Hughes Medical Institute, Chevy Chase, MD USA

**Keywords:** Cell biology, Biological techniques

## Abstract

The regulation of metabolic processes by proteins is fundamental to biology and yet is incompletely understood. Here we develop a mass spectrometry (MS)-based approach that leverages genetic diversity to nominate functional relationships between 285 metabolites and 11,868 proteins in living tissues. This method recapitulates protein–metabolite functional relationships mediated by direct physical interactions and local metabolic pathway regulation while nominating 3,542 previously undescribed relationships. With this foundation, we identify a mechanism of regulation over liver cysteine utilization and cholesterol handling, regulated by the poorly characterized protein LRRC58. We show that LRRC58 is the substrate adaptor of an E3 ubiquitin ligase that mediates proteasomal degradation of CDO1, the rate-limiting enzyme of the catabolic shunt of cysteine to taurine^1^. Cysteine abundance regulates LRRC58-mediated CDO1 degradation, and depletion of LRRC58 is sufficient to stabilize CDO1 to drive consumption of cysteine to produce taurine. Taurine has a central role in cholesterol handling, promoting its excretion from the liver^2^, and we show that depletion of LRRC58 in hepatocytes increases cysteine flux to taurine and lowers hepatic cholesterol in mice. Uncovering the mechanism of LRRC58 control over cysteine catabolism exemplifies the utility of covariation MS to identify modes of protein regulation of metabolic processes.

## Abstract (web summary)

A mass spectrometry-based approach globally identifies protein regulators of metabolism and reveals the role of LRRC58 in controlling cysteine catabolism.

## Main

Metabolic reactions are executed by proteins and metabolites. Regulation between these classes of molecules is often low affinity or not based on direct physical interactions, making these phenomena challenging to investigate. Hypothesis-driven studies have so far explored coordination between individual proteins and metabolites^3,4^. More recently, elegant techniques have been developed to study these regulatory events on a larger scale^4–8^. Most of these approaches are limited by the availability of recombinant proteins and/or pure metabolites, and examination is under non-native conditions. Moreover, current methods do not capture modes of regulation that are not based on direct physical interactions between individual metabolites and proteins. This form of metabolic regulation is prevalent in biological systems^9^, including functional relationships that occur on a pathway level in vivo.

We recently developed an approach to identify co-operative functions between proteins in living tissues^10^. This method relies on analysis of abundance covariation between proteins in a genetically defined diversity outbred mouse model (DO mice) that parallels the genetic and phenotypic variability found in humans^11^. Here we evolve this method by combining protein and metabolite MS to investigate functional relationships between 285 metabolites and 11,868 proteins in living tissues. Using these data, we develop a metabolite–protein covariation architecture (MCPA). MPCA is a machine learning approach we use to nominate 3,542 previously uncharacterized metabolite–protein functional relationships. MPCA is provided as an online resource at https://mpca-chouchani-lab.dfci.harvard.edu/.

With this method as a foundation, we sought to uncover mechanisms of control over disease-relevant metabolic processes. We focused our attention on a poorly understood aspect of cellular metabolism: regulation of cysteine catabolism. Using MPCA, we discover a protein complex that responds to cysteine abundance to regulate its catabolism to taurine, facilitated by the protein LRRC58.

## Building MPCA in living mouse tissues

**Fig. 1:**
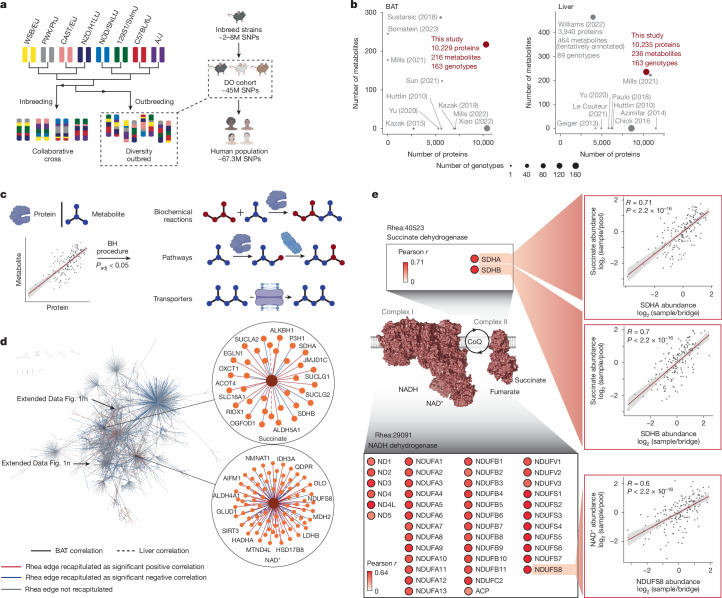
Protein–metabolite covariation in the DO cohort recapitulates established biochemical reactions. **a**, Breeding scheme and genetic diversity of the DO cohort. SNPs, single nucleotide polymorphisms. Created in BioRender. Xiao, H. (2025) https://BioRender.com/cluhh92. **b**, BAT and liver, two metabolically heterogenous tissues, were selected for deep proteomics and metabolomic profiling. The figure shows proteins and metabolites measured from BAT and liver of different genotypes of mice in this work alongside those from previous studies^10,12,16–28,60^. *n* = 163 mice. **c**, Abundance correlation between individual proteins and metabolites in each tissue were filtered using the Benjamini–Hochberg (BH) procedure, and then used to recapitulate established biochemical reactions, pathways and transporter–metabolite relationships. Details in Methods. *P*_adj_, adjusted *P* value. **d**, Overview of Rhea edge recapitulation analysis. The entire Rhea reaction network mapped in MPCA is illustrated. Each metabolite–enzyme interaction is shown as an edge between a metabolite and a protein node. Edges between succinate and NAD^+^ and proteins are magnified. *n* = 163 mice. See Supplementary Table 3 for the underlying dataset. **e**, MPCA edges recapitulate relationships between succinate, NAD^+^ and mitochondrial electron transport chain proteins. Two-sided Pearson correlation test with Benjamini–Hochberg *P*-value correction. Error band represents the 95% confidence interval.

The principle of defining functional relationships between biomolecules through co-regulation relies on assessing correlations in abundance across a heterogeneous population^10,12^. This approach has been applied for identification of coordinated protein function^10^. Here we explored whether the same principle could be applied to functional relationships between metabolites and proteins. To build MPCA, we used a cohort of 163 fully genotyped female DO mice, which exhibit a high degree of genetic, proteomic and phenotypic heterogeneity that approaches that of the human population^11,13^ (Fig. 1a). To further exploit this principle of co-operativity, we analysed two tissues that exhibit substantial inter-individual metabolic variability and capture a wide range of metabolic activities. We selected liver and brown adipose tissue (BAT), as these tissues capture major metabolic processes that are relevant to mammalian physiology^14,15^.

**Extended Data Fig. 1:**
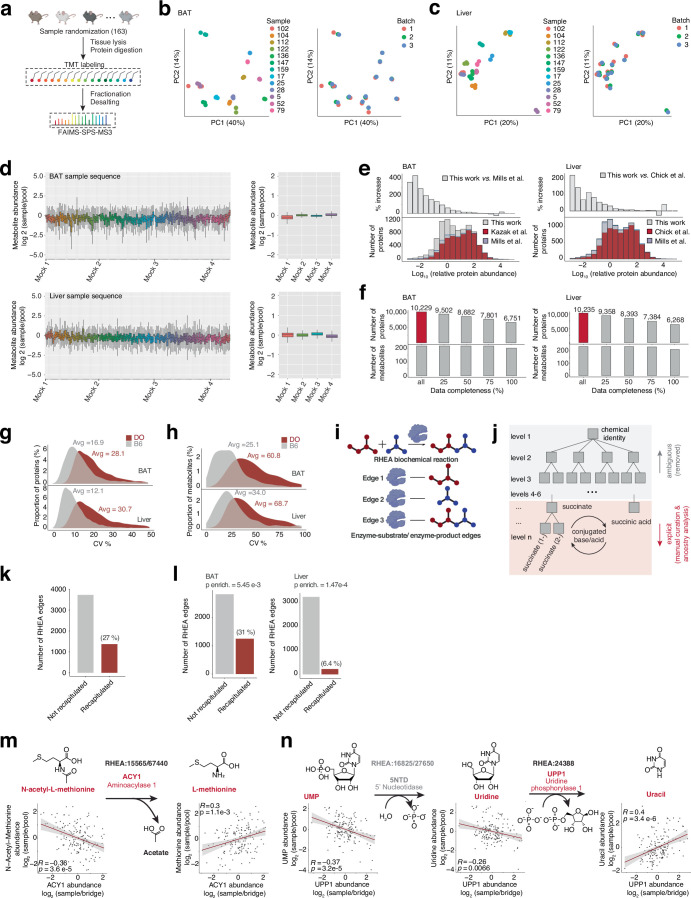
MPCA technical quality evaluation and recapitulation of RHEA reactions. (**a**) Sample preparation workflow for DO proteomics. Created in BioRender. Xiao, H. (2025) https://BioRender.com/c8oe3gt. (**b**) PCA plots showing samples from repeated proteomics measurements in BAT, colored by sample ID and measurement batch. (**c**) PCA plots showing samples from repeated proteomics measurements in liver, colored by sample ID and measurement batch. (**d**) Overview of relative metabolite abundance (log_2_ sample-bridge ratio) in both BAT and liver of all samples measured in this study. Mock samples were the same as pools, which were spiked in every ~50 runs in the sequence to assess instrument performance (see Methods for details). The center of the boxplot is median, the box bounds show the 25th to 75th percentile interquartile range (IQR), and the minimum and maximum values are 1.5 times the IQR. n = 163 mice. (**e**) The proteome depth in MPCA compared to previous deep mouse BAT and liver proteomics reports^12,19,21,22^. (**f**) Proteomics and metabolomics data completeness in BAT and liver. (**g**) DO cohort exhibit higher protein abundance variation proteome-wide in BAT and liver compared to isogenic cohorts. C57BL/6 data obtained from Yu et al.^27^. (**h**) DO cohort exhibited higher metabolite abundance variation in BAT and liver compared to isogenic cohorts. BAT C57BL/6 data obtained from Jung et al.^29^, liver C57BL/6 data obtained from Xiao et al.^63^. (**i**) Enzyme-substrate and enzyme-product edges generated from RHEA. (**j**) Ancestry analysis (see Methods for details). All metabolites were annotated by the following Chemical Entities of Biological Interest (ChEBI) IDs: the ChEBI IDs of the metabolite itself; the ChEBI IDs of its conjugate acids and/or bases; the ChEBI IDs of its salt adducts; the ChEBI IDs of the metabolite with different charge states; and ChEBI IDs of chemicals that have the same chemical formula and structure but named differently. Both primary and secondary ChEBI IDs were extracted. These IDs were then used for ancestry mapping using the ancestry mapping table provided by ChEBI, in order to match a subset of entries in databases that do not use IDs at the bottom of the ChEBI hierarchy. The first 6 levels of IDs in the ChEBI hierarchy were not used for ancestry mapping due to the ambiguous nature. This analysis was to prepare metabolites for mapping onto established databases, as ChEBI IDs are universal metabolite identifiers for most databases. (**k**) MPCA edges recapitulate 27% of all RHEA edges involving molecules measured in MPCA. (**l**) Recapitulation of established biochemical reactions using significant metabolite-protein correlations in BAT and liver. (**m**) MPCA recapitulation of RHEA reaction 15565 and 67440, deacylation of N-acetyl-L-methionine. n = 163 mice. (**n**) MPCA recapitulation of RHEA reaction 24388, dephosphorylation of uridine and its subsequent conversion to uracil. RHEA reaction 16825/27650 were indirectly recapitulated. n = 163 mice. (One-sided Fisher’s exact test in l, two-sided Pearson correlation test with Benjamini-Hochberg p value correction in m, n; error bands in m and n represent 95% confidence interval.).

We began by quantifying 11,868 proteins and 285 metabolites in BAT and liver from each individual in the DO cohort (Supplementary Table 1). All samples were randomized, and proteomic and metabolomic measurements exhibited low technical variability and no observable batch effects (Extended Data Fig. 1a–d). With more 3.4 million molecular measurements from BAT and liver of this cohort, the coverage of this work greatly exceeded those in previous reports^10,12,16–28^, especially in measuring low-abundance proteins (Fig. 1b and Extended Data Fig. 1e). Proteins and metabolites were quantified with high data completeness (Extended Data Fig. 1f). Unlike isogenic cohorts^27,29^, the genetic diversity of the DO cohort led to substantial proteomic and metabolomic variations (Extended Data Fig. 1g,h), which enabled us to derive tissue-specific covariations and examine coordinated metabolic processes (Fig. 1c). In total, MPCA queried 482,043 non-redundant and statistically significant correlation pairs (5% false discovery rate (FDR)), including in both tissues a total of 134,740 co-operative relationships and 359,062 antagonistic relationships (Supplementary Table 2).

## Metabolite–protein relationships in MPCA

Protein abundance correlations have previously been used to assess co-operative functions between proteins^10,30,31^. Whether abundance covariation between proteins and metabolites in outbred cohorts would reflect functional relationships is not known. To examine this, we explored whether MPCA-derived correlations recapitulated known functional relationships between metabolites and proteins.

## Direct metabolite–protein interactions

We first examined direct physical relationships by mapping all significant protein–metabolite correlations onto Rhea^32^. We converted Rhea reactions into protein–metabolite pairs to represent all known enzyme–substrate or enzyme–product relationships in human and mouse biology (Extended data Fig. 1i–k and Supplementary Table 3). A total of 27% (*n* = 1,373) of Rhea enzyme–substrate or enzyme–product relationships that were mapped in our dataset were recapitulated by MPCA as statistically significant metabolite–protein co-operative pairs (Fig. 1d, Extended Data Fig. 1i–l and Supplementary Table 3). MPCA captured established metabolite–protein relationships that encompass major aspects of cellular metabolism, including components of the mitochondrial electron transport chain, amino acid metabolism and nucleotide metabolism (Fig. 1e and Extended Data Fig. 1m,n; further discussed in Supplementary Discussion).

## Protein transporters of metabolites

**Extended Data Fig. 2:**
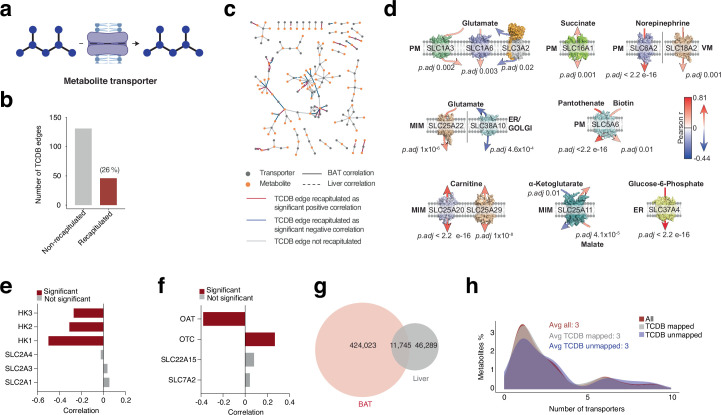
MPCA edges recapitulate metabolite-transporter relationships. (**a**) Metabolite-transporter relationship as a form of metabolite-protein co-regulation. (**b**) MPCA edges recapitulate 26% of all protein-metabolite edges in the transporter classification database (TCDB) involving molecules measured in MPCA. (**c**) Visualization of TCDB transporter-metabolite relationships recapitulated by MPCA edges. (**d**) Examples of TCDB transporter-metabolite relationships recapitulated by MPCA edges. (**e**) Pairwise correlations between glucose and HK1, HK2, HK3, SLC2A1, SLC2A3, and SLC2A4. (**f**) Pairwise correlations between ornithine and OAT, OTC, SLC22A15, and SLC7A2. (**g**) Shared and unique protein-metabolite edges in BAT and liver. (**h**) The average number of TCDB transporters each metabolite has. All- all metabolites; TCDB mapped- metabolites that have their TCDB transporters mapped in MPCA; TCDB-unmapped- metabolites that did not have their TCDB transporters mapped in MPCA.

Metabolite transporters can have central roles in determining local metabolite abundance. To examine whether MPCA could identify metabolite–transporter relationships, we mapped significant correlations in MPCA onto the Transporter Classification Database (TCDB)^33^ (Extended Data Fig. 2a and Supplementary Table 3). In total, 26 % (*n* = 46) of all known transporter–substrate relationships involving the measured molecules were recapitulated by MPCA (Extended Data Fig. 2b–h). Additional analyses of these correlations are provided in the Supplementary Discussion.

## Pathway-level co-operativity

**Extended Data Fig. 3:**
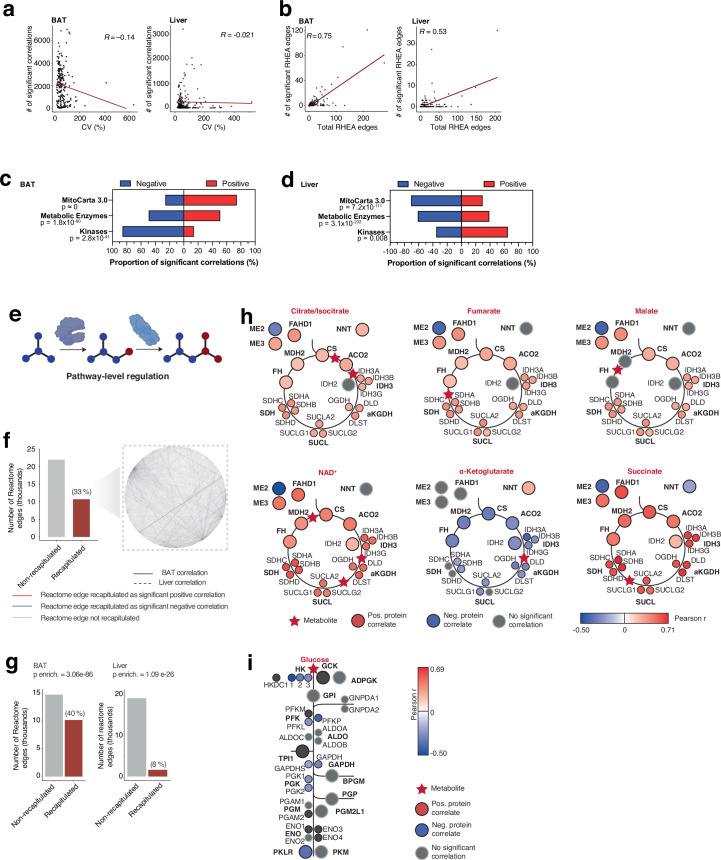
Analysis of factors that underlie significant protein-metabolite correlations in MPCA. (**a**) Correlation between the number of significant correlations between each MPA metabolite with proteins and the degree of variation that this metabolite exhibited in the cohort. (**b**) Correlation between the number of significant RHEA edges and the number of total RHEA edges of a metabolite in MPCA. (**c**) Enrichment of mitochondrial proteins, metabolite enzymes, and kinases among protein nodes of all MPA edges in BAT. (**d**) Enrichment of mitochondrial proteins, metabolite enzymes, and kinases among protein nodes of all MPCA edges in liver. (**e**) Pathway-level regulation as a form of metabolite-protein functional co-regulation. (**f**) MPCA edges recapitulated 33% of all protein-metabolite edges in Reactome involving molecules measured in MPCA. (**g**) Recapitulation of established metabolite-protein relationships in Reactome pathways using significant metabolite-protein correlations in BAT and liver. (**h**) Correlation between the abundance of TCA cycle intermediary metabolites succinate, α-ketoglutarate, citrate/isocitrate, fumarate, malate, as well as co-factor NAD^+^, with TCA cycle enzymes. (**i**) Glucose abundance negatively correlated with the abundance of glycolysis enzymes. (One-sided Fisher’s exact test in c, d, and g, P values were not adjusted).

**Extended Data Fig. 4:**
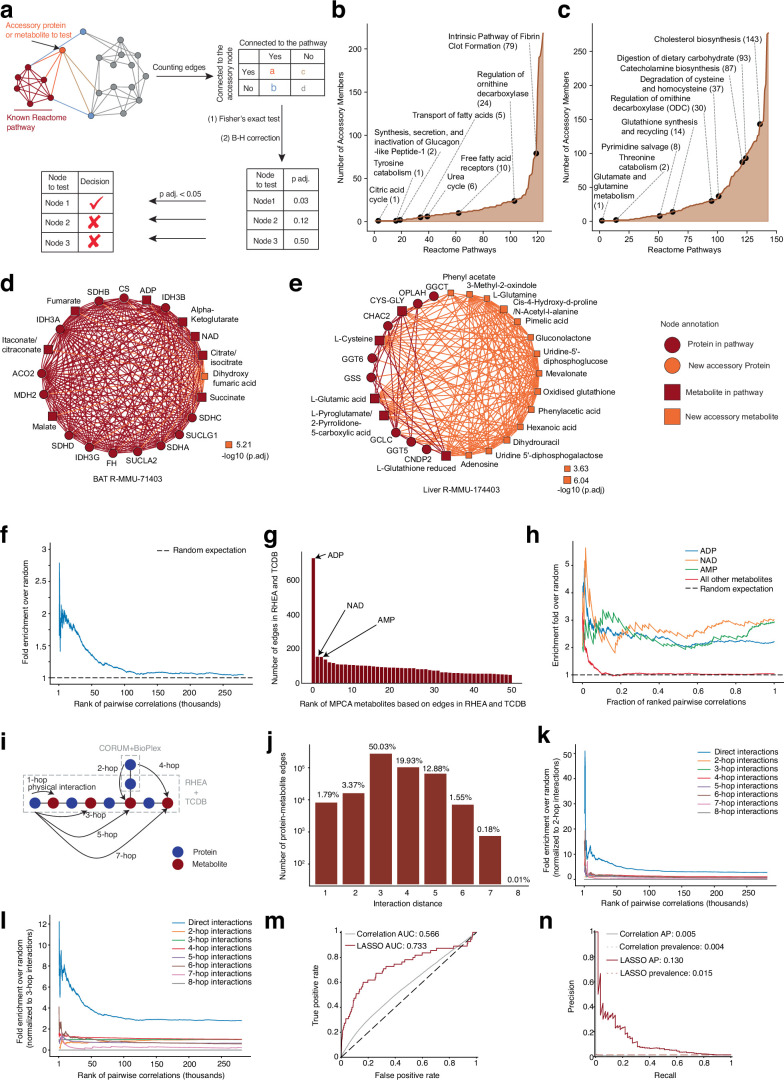
Statistical properties of pairwise correlations in MPCA. (**a**) Linking accessory proteins and metabolites of known Reactome pathways. Red nodes, known members of established networks; blue nodes, neighboring proteins or metabolites; orange node, the protein or metabolite to test; gray nodes, all other proteins and metabolites in MPCA. (**b**) Reactome pathways and newly identified accessory proteins in BAT. (**c**) Reactome pathways and newly identified accessory proteins in liver. (**d**) TCA cycle with an accessory metabolite in BAT. (**e**) Glutathione synthesis and recycling pathway with accessory members in liver. (**f**) Fold enrichment over random selection of MPCA pairwise correlations recapitulating physical interactions in RHEA and TCDB. MPCA correlations were filtered to contain proteins and metabolites existing in RHEA and TCDB. (**g**) Rank of metabolites measured in MPCA based on the number of protein-metabolite intractions in RHEA and TCDB. (**h**) Fold enrichment over random selection of MPCA edges involving ADP, NAD, and AMP recapitulating physical interactions in RHEA and TCDB, compared to all other edges. (**i**) Schematic of “hop” analysis to examine physical interactions and non-specific interactions underlying protein-metabolite correlations in MPCA. (**j**) The number of protein-metabolite edges included in each “hop”, 1-hop means physical interactions. The percentage annotates proportion of all possible protein-metabolite edges. (**k**) Fold enrichment over random selection of MPCA edges recapitulating physical interactions in RHEA and TCDB, compared to non-specific interactions introduced from the “hop” analysis. Fold enrichment normalized to 2-hop. (**l**) Fold enrichment over random selection of MPCA edges recapitulating physical interactions in RHEA and TCDB, compared to non-specific interactions introduced from the “hop” analysis. Fold enrichment normalized to 3-hop. (**m**) ROC curves of pairwise correlation analysis (gray), LASSO analysis (red, referring to Fig. 2a), and null distribution (black). AUC- area under the curve. (**n**) PR curves of pairwise correlation analysis (gray), LASSO analysis (red, referring to Fig. 2a). AP- average precision. (One-sided Fisher’s exact test with Benjamini-Hochberg p value correction in d,e).

We next investigated underlying factors that determine the number of significant co-operative and antagonistic interactions for each metabolite in MPCA. The number of protein co-variates with each metabolite did not correlate with the degree of metabolite abundance variation across the DO cohort (Extended Data Fig. 3a). Instead, the number of recapitulated metabolite–protein relationships was positively associated with the number of established biochemical reactions linked to each metabolite (Extended Data Fig. 3b). This suggests that in MPCA, metabolites with higher numbers of protein correlates participate in more biological reactions, serving as substrates, products or cofactors. A prominent example is NAD^+^, which is a critical redox equivalent and electron carrier used in many biochemical reactions (Fig. 1d,e). We further analysed co-operativity at the pathway level (Extended Data Figs. 3c–i and 4a–e and Supplementary Table 4) and statistical properties of covariation derived from different forms of metabolite–protein relationships (Extended Data Fig. 4f–n). These analyses are provided in the Supplementary Discussion.

## Systematic discovery using MPCA

**Fig. 2:**
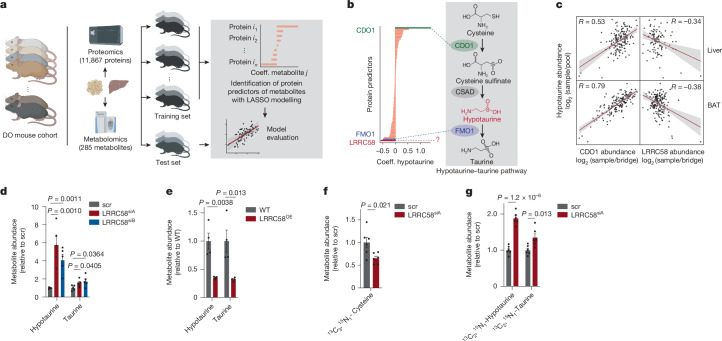
MPCA identifies LRRC58 as a negative regulator of hypotaurine and taurine production. **a**, Schematic of machine learning based on LASSO regression to identify protein regulators of metabolites. Coeff., coefficient. Created in BioRender. Xiao, H. (2025) https://BioRender.com/lpjycc1. **b**, LASSO regression identified proteins that predict hypotaurine abundance in BAT. *n* = 163 mice. **c**, Correlation between CDO1 abundance and hypotaurine abundance, as well as correlation between LRRC58 abundance and hypotaurine abundance, in liver and BAT. Liver, *n* = 162 mice; BAT, *n* = 163 mice. **d**, Comparison of hypotaurine and taurine in scr and LRRC58^KD^ (induced by siRNA A (LRRC58^siA^) or siRNA B (LRRC58^siB^)) Hep G2 cells. *n* = 4 cell replicates. Data replotted from Extended Data Fig. 6g. **e**, Comparison of hypotaurine and taurine in wild-type (WT) and LRRC58^OE^ Hep G2 cells. *n* = 4 cell replicates. Underlying data replotted from Extended Data Fig. 6i. **f**, Comparison of ^13^C_3_^15^N_1_-l-cysteine abundance in scramble and LRRC58^siA^ primary hepatocytes following 30 min incubation with 200 µM ^13^C_6_^15^N_2_-labelled l-cystine. *n* = 6 cell replicates. **g**, Comparison of ^13^C_2_^15^N_1_-hypotaurine and ^13^C_2_^15^N_1_-taurine abundance in scr and LRRC58^siA^ primary hepatocytes following 30 min incubation with 200 µM ^13^C_6_^15^N_2_-labelled l-cystine. *n* = 6 cell replicates. Two-tailed Student’s *t*-test for pairwise comparisons (**d**–**g**). Data are mean ± s.e.m.; error band in **c** represents the 95% confidence interval.

To leverage MPCA to identify previously unknown protein–metabolite relationships, we used a regression model based on least absolute shrinkage and selection operator (LASSO)^34^ to rank functional relationships between metabolites and proteins in MPCA (Fig. 2a and Methods). LASSO analysis penalizes proteins with only minor contributions in determining metabolite abundance by assigning a coefficient of zero, which nominates proteins that can be prioritized for biological validation. This modelling approach led to annotation of 3,542 total protein predictors of metabolite abundance with non-zero prediction coefficients in BAT and liver that have not been described previously (Supplementary Table 5).

## Statistical properties of MPCA LASSO

**Extended Data Fig. 5:**
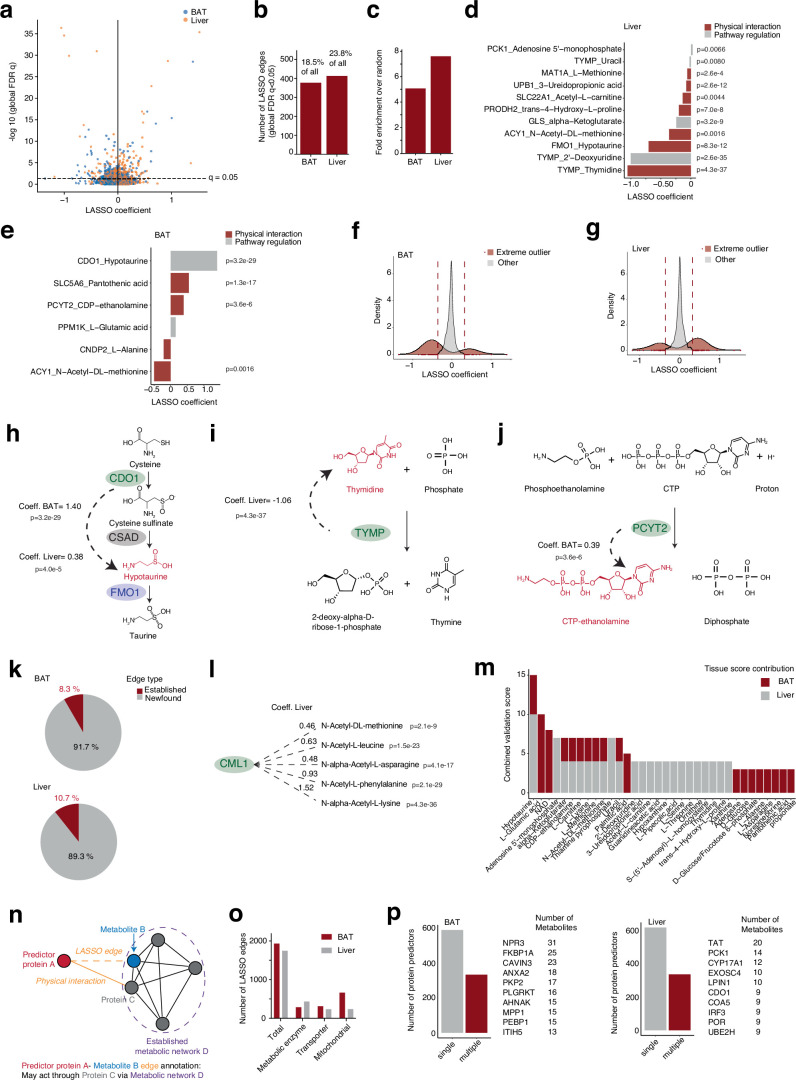
Statistical properties of LASSO analysis in MPCA. (**a**) Evaluation of LASSO associations with global FDR q < 0.05. P-values were calculated by performing ordinary least squares (OLS) on those selected variables from LASSO, and p-values were computed from the OLS model using a two-sided t-test. FDR values were calculated by adjusting P-values with the Benjamini-Hochberg procedure. FDR q < 0.05 was considered significant. (**b**) Number and percentage of LASSO associations in BAT and liver that reached global FDR q < 0.05. (**c**) Enrichment over random selection of significant LASSO associations in BAT and liver in recapitulating physical interactions between proteins and metabolites in RHEA and TCDB. (**d**) Top1 LASSO predictors of metabolites with literature evidence in liver. (**e**) Top1 LASSO predictors of metabolites with literature evidence in BAT. (**f**) Extreme outliers in LASSO analysis identified in BAT. (**g**) Extreme outliers in LASSO analysis identified in liver. (**h**) LASSO Coefficient of the CDO1-hypotaurine edge. (**i**) LASSO Coefficient of the TYMP-thymine edge. (**j**) LASSO Coefficient of the PCY2-CTP-ethanolamine edge. (**k**) Percent of extreme outliers in BAT and liver with literature evidence. (**l**) Extreme outlier edges involving CML1, ACY3, and acetylated amino acids. (**m**) Validation score of metabolites in MPCA. For each metabolite, LASSO edges with literature evidence in RHEA, TCDB, and Reactome were counted. This was then used to linearly scale, in each tissue, to a score from 1–10 based on the number of recapitulated LASSO edges all the other metabolites have. The score from both tissues were then summed up to produce an overall validation score for each metabolite. (**n**) Annotation of putative function of LASSO protein predictors of metabolites. LASSO hits for each metabolite were mapped onto CORUM^35^ and BioPlex^36^. If a newfound LASSO protein predictor of a metabolite physically interacts with a protein known to regulate this metabolite via a known RHEA, TCDB, or Reactome network, then the LASSO hit was listed as potentially regulating the metabolite through the known network (Supplementary Table 5c). (**o**) Annotation of LASSO protein predictors of metabolites based on whether the proteins were known to be metabolic enzymes, transporters, and mitochondrial proteins. (**p**) Top 10 proteins in BAT and liver that predicted the highest number of metabolites. (OLS modeling for selected LASSO variables and two-sided t-test to calculate P values. P values adjusted by the Benjamini-Hochberg procedure in a, d, e, h-j, l).

We first evaluated global statistical significance and enrichment across LASSO predictions in each tissue. Among all non-zero LASSO protein–metabolite associations, 771 were statistically significant with a global FDR < 0.05, 18.5% and 23.8% of all associations in BAT and liver, respectively (Extended Data Fig. 5a,b and Supplementary Table 5). These LASSO predictions reported established physical interactions in Rhea and TCDB with a fold enrichment over random discovery of 5.06 in BAT and 7.52 in liver (Extended Data Fig. 5c). We then rank-ordered LASSO protein predictors for each metabolite on the basis of absolute value of the coefficients, as these coefficients represent the contribution of each protein to metabolite abundance prediction. We examined the extent to which top-1 protein predictors of metabolite abundance recapitulated known direct and local regulators of metabolite abundance. We found that in liver, 113 metabolites had non-zero LASSO predictors, and 11 metabolites had their top-ranked abundance predictors already established in the literature (Extended Data Fig. 5d). In BAT, 132 metabolites had non-zero abundance predictors, and 6 metabolites had top-1 predictors already established in the literature (Extended Data Fig. 5e). These demonstrated that LASSO analysis was able to nominate local protein regulators of metabolite abundance.

We further extended this analysis to protein predictors that were extreme outliers. We identified 135 extreme outliers on the basis of interquartile range (IQR), and points beyond 3× IQR below quartile 1 (Q1) or above quartile 3 (Q3) were determined to be extreme outliers (Extended Data Fig. 5f,g). Thirteen out of 135 extreme outlier edges were known to regulate the metabolite being predicted within the local metabolic network (examples provided in Extended Data Fig. 5h–j). A total of 8.3% of extreme outlier edges in BAT and 10.7% in liver contained established protein–metabolite relationships based on Rhea, TCDB and Reactome (Extended Data Fig. 5k). We used precision-recall and receiver operating characteristic (ROC) curves to assess the predictive ability of LASSO analysis (Extended Data Fig. 4m,n). Compared with pairwise correlation, LASSO modelling significantly improved performance (Extended Data Fig. 4m,n). Of note, these analyses likely underestimated the true performance since Rhea, TCDB and Reactome do not capture all known relationships between metabolites and proteins. For instance, extreme outlier edges identified many biologically plausible protein regulators of metabolite abundance that were not present in the aforementioned reference databases. An example is CML1, the homologue of NAT8, a human *N*-acetyltransferase. Although biochemical characterization of the acetyltransferase activity of CML1 is lacking, it is an extreme outlier for *N*-acetyl-dl-methionine, *N*-acetyl-l-leucine, *N-*α-acetyl-l-asparagine, *N*-acetyl-l-phenylalanine and *N-*α-acetyl-l-lysine (Extended Data Fig. 5l), suggesting that CML1 may be a ubiquitous acetyltransferase in mice. Although ground truth datasets were incomplete, these analyses indicated the utility of LASSO modelling for the identification of previously uncharacterized functional protein–metabolite relationships (Extended Data Fig. 4m,n).

On the basis of the above analyses, we generated a validation score for each metabolite (Extended Data Fig. 5m) to prioritize the selection of metabolites for validating newly identified protein–metabolite relationships. We assessed each metabolite by considering the presence of known physical interactors, local pathway regulators and transporters in their non-zero protein predictor list. Metabolites with a higher number of known protein predictors received a correspondingly higher validation score (Extended Data Fig. 5m and Supplementary Table 5). To systematically explore the function of protein LASSO predictors of metabolites, we mapped all LASSO hits for each metabolite onto the Comprehensive Resource of Mammalian Protein Complexes (CORUM)^35^ and BioPlex^36^. If a newfound LASSO protein predictor of a metabolite physically interacts with a protein that is known to regulate this metabolite via a local metabolic network, we annotate the LASSO hit as potentially regulating the metabolite through the known network (Extended Data Fig. 5n and Supplementary Table 5). Through this approach, we were able to nominate putative functions of 136 protein predictors of metabolites. In addition, we annotated LASSO protein–metabolite associations on the basis of whether the protein was a known metabolic enzyme or transporter, or was associated with mitochondrial function (Extended Data Fig. 5o and Supplementary Table 5). In total, 1,477 (40.1%) of all LASSO edges were annotated on the basis of putative or established function of the protein, of which 65 were extreme outliers (48.1% of all extreme outliers). We expect that extreme outliers that have putative functional annotations and are associated with metabolites with high validation scores will be most actionable for downstream functional validation.

We also determined and annotated proteins that predicted the abundance of multiple metabolites (Extended Data Fig. 5p). These proteins are more likely to regulate upstream metabolic processes for some of the metabolites being predicted, rather than being confined to the local metabolic network of a predicted metabolite.

## LRRC58 control of hypotaurine

We next sought to use MPCA to uncover mechanisms of control over disease-relevant metabolic processes. We focused our attention on the metabolic shunt that catabolizes cysteine to hypotaurine and taurine^37^. This pathway regulates cysteine abundance in cells, which is pivotal for regulation of glutathione homeostasis^38^, iron metabolism^39^ and cysteine toxicity^40^. Flux of cysteine through this shunt is highly regulated, and hypotaurine and taurine are among the most abundant endogenous metabolites^41,42^. Moreover, taurine is known to conjugate bile acids produced by cholesterol in the liver, which promotes both cholesterol and bile acid excretion^2^.

The rate-limiting enzyme for cysteine conversion to hypotaurine and taurine is CDO1^1,43^. How the conversion of cysteine to taurine is regulated remains unknown, but appears to be attributable at least in part to post-transcriptional control of CDO1 abundance through an undefined mechanism^44^. CDO1 abundance has been implicated in determining cellular abundance of cysteine and taurine, and in regulation of cholesterol homeostasis in liver^2,38,43,45^. Since cysteine conversion to taurine appears to be tightly regulated through an unknown post-transcriptional mechanism involving CDO1^44^, we explored whether MPCA could uncover the mechanisms underlying this process.

**Extended Data Fig. 6:**
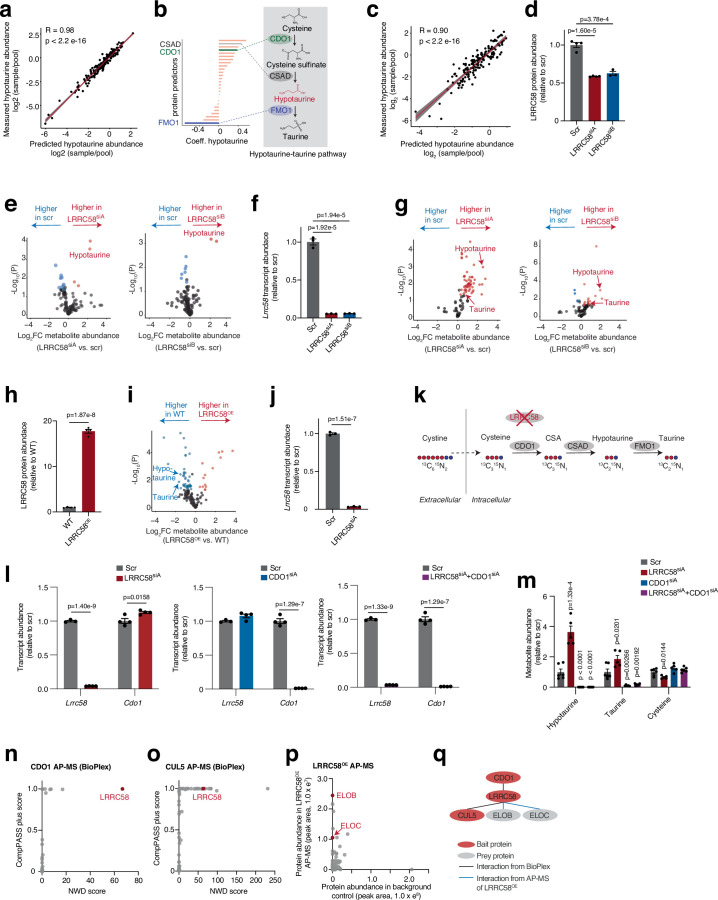
Genetic manipulation of LRRC58 in the context of hypotaurine-taurine pathway. (**a**) Correlation between hypotaurine abundance calculated from LASSO prediction and hypotaurine abundance measured in BAT. n = 163 mice. (**b**) LASSO regression to predict hypotaurine abundance in the liver. CDO1- cysteine dioxygenase type 1; CSAD - cysteine sulfinic acid decarboxylase; FMO1- flavin containing monooxygenase 1. LRRC58 was not included in liver analysis due to its low abundance leading to 59 missing values out of 163 samples in total. Hypotaurine measurement was missing in the liver of one mouse. n = 162 mice. (**c**) Correlation between hypotaurine abundance calculated from LASSO prediction and hypotaurine abundance measured in liver. n = 162 mice. (**d**) Protein abundance of LRRC58 in scramble (scr) compared to LRRC58 siRNA A (LRRC58^siA^) and siRNA B (LRRC58^siB^)-treated primary brown adipocytes. n = 4 cell replicates for scr and LRRC58^siA^; n = 3 cell replicates for LRRC58^siB^. Data replotted from Fig. 3a. (**e**) Left: metabolite abundance profiling comparing scramble (scr) to LRRC58 siRNA A (LRRC58^siA^)-treated primary brown adipocytes. n = 4 cell replicates. Right: metabolite abundance profiling comparing scramble (scr) to LRRC58 siRNA B (LRRC58^siB^)-treated primary brown adipocytes. n = 4 cell replicates. (**f**) Transcript abundance of *Lrrc58* in scramble (scr) compared to LRRC58^siA^ and LRRC58^siB^-treated primary hepatocytes. n = 3 cell replicates. (**g**) Left: metabolite abundance profiling comparing scramble (scr) to LRRC58 siRNA A (LRRC58^siA^)-treated primary hepatocytes. n = 4 cell replicates. Left: metabolite abundance profiling comparing scramble (scr) to LRRC58 siRNA B (LRRC58^siB^)-treated primary hepatocytes. n = 4 cell replicates. (**h**) Protein abundance of LRRC58 in wild type (WT) compared LRRC58 overexpression (LRRC58^OE^) in Hep G2. n = 4 cell replicates. Data replotted from Fig. 3e. (**i**) Metabolite abundance profiling comparing wildtype (WT) to LRRC58 overexpression (LRRC58^OE^) Hep G2 cells. n = 4 cell replicates. (**j**) Transcript abundance of *Lrrc58* in scramble (scr) compared to LRRC58 siRNA A (LRRC58^siA^)-treated primary hepatocytes for the tracing experiments. n = 3 cell replicates. (**k**) Schematic of tracing cysteine metabolism to taurine in scramble (scr) and LRRC58^KD^ primary hepatocytes. Cells were treated for 30 min with 200 µM ^13^C_6_^15^N_2_-labeled L-cystine. ^13^C_3_^15^N_1_-L-cysteine, ^13^C_2_^15^N_1_-hypotaurine, and ^13^C_2_^15^N_1_-taurine are the expected major forms of isotope-labeled downstream metabolites in the hypotaurine-taurine pathway. (**l**) Transcript abundance of *Lrrc58* and *CDO1* in scramble (scr) compared to LRRC58^siA^, CDO1^siA^, and LRRC58^siA^ + CDO1^siA^ -treated primary hepatocytes. n = 4 cell replicates. (**m**) Hypotaurine, taurine and L-cysteine abundance in scramble (scr) compared to LRRC58^siA^, CDO1^siA^, and LRRC58^siA^ + CDO1^siA^ -treated primary hepatocytes. n = 6 cell replicates. (**n**, **o**) AP-MS of CDO1 and CUL5 from BioPlex 3.0^36^. CompPASS plus score ranges from 0-1, and a score of 1 represents the highest confidence for identification of a physical interactor. Normalized weighted D (NWD) score takes account of the selectivity of the interaction. A higher NWD score indicates higher selectivity of the interaction. LRRC58 was identified in only 5 out of all 10,128 experiments in BioPlex 3.0. n = 2 technical replicates. (**p**) AP-MS of LRRC58 in LRRC58 overexpression (LRRC58^OE^) Hep G2 cells (see Methods). Protein abundance presented by summed MS1 peak area. n = 4 cell replicates for LRRC58^OE^. n = 1 cell replicate for background control. (**q**) Interactions between LRRC58, CDO1, CUL5, ELOB, and ELOC based on literature evidence and experimental data. (two-sided Pearson correlation test in a with no multiple comparison adjustment; two-tailed Student’s t-test for pairwise comparisons in d-j, l, and m with no multiple comparison adjustment. P < 0.05 considered significant). Data presented as mean ± s.e.m., error band in a and c represent 95% confidence interval.

MPCA identified 81 proteins whose abundance collectively predicted the abundance of hypotaurine in BAT with high prediction accuracy (Fig. 2b and Extended Data Fig. 6a). The protein that most positively predicted hypotaurine abundance was CDO1, confirming its high degree of control over hypotaurine biosynthesis (Fig. 2b). Conversely, FMO1, the protein that consumes hypotaurine to produce taurine^46^, strongly negatively predicted hypotaurine abundance (Fig. 2b). These proteins also predicted hypotaurine abundance in the liver (Extended Data Fig. 6b,c). Notably, MPCA also identified LRRC58, a protein with no established metabolic role in mouse or human cells, as the strongest negative contributor to hypotaurine abundance (Fig. 2b,c). We therefore proceeded to explore the relevance of LRRC58 in this pathway.

## LRRC58 antagonizes cysteine catabolism

We began with knockdown of LRRC58 in primary brown adipocytes, which led to a fivefold increase in hypotaurine abundance compared with wild-type cells (Extended Data Fig. 6d,e and Supplementary Table 6). In primary hepatocytes, knockdown of LRRC58 increased hypotaurine abundance by approximately fivefold and taurine abundance by 75% (Fig. 2d and Extended Data Fig. 6f,g). Conversely, stable overexpression of LRRC58 in Hep G2 liver cells depleted hypotaurine and taurine (Fig. 2e and Extended Data Fig. 6h,i). We next examined whether LRRC58 regulated the abundance of these metabolites by affecting their production from cellular cysteine. Tracing labelled cystine in primary hepatocytes revealed that depletion of LRRC58 drove increased flux from cysteine to hypotaurine and taurine (Fig. 2f,g and Extended Data Fig. 6j,k).

**Fig. 3:**
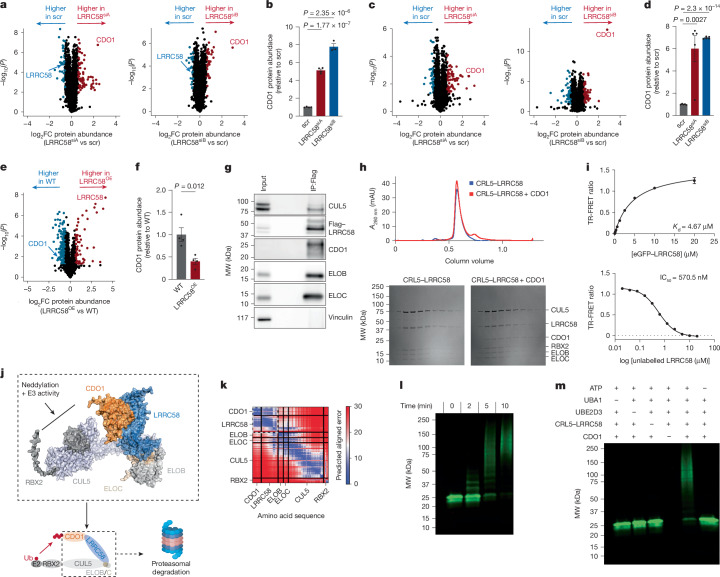
LRRC58 is a substrate adaptor for an E3 ligase that targets CDO1 for degradation. **a**, Proteomics analysis comparing scr to LRRC58^siA^ (left) and LRRC58^siB^ (right) primary brown adipocytes. LRRC58^siB^, *n* = 3; other groups cell replicates, *n* = 4 cell replicates. **b**, Comparison of CDO1 abundance in scr, LRRC58^siA^ and LRRC58^siB^ primary brown adipocytes. LRRC58^siB^, *n* = 3 cell replicates; other groups, *n* = 4 cell replicates. Data replotted from **a**. **c**, Proteomics analysis comparing scr to LRRC58^siA^ and LRRC58^siB^ primary hepatocytes. *n* = 5 cell replicates. **d**, Comparison of CDO1 abundance in scr, LRRC58^siA^ and LRRC58^siB^ primary hepatocytes. *n* = 5 cell replicates. Data replotted from **c**. **e**, Proteomics analysis comparing WT to LRRC58^OE^ Hep G2 cells. *n* = 4 cell replicates. **f**, Comparison of CDO1 abundance in WT (scr) and LRRC58^OE^ Hep G2 cells. *n* = 4 cell replicates. Data replotted from **e**. **g**, Flag immunoprecipitation (IP) followed by western blotting from Hep G2 cells expressing Flag-tagged LRRC58. *n* = 3 cell replicates. **h**, Top, size-exclusion chromatography (SEC) of CRL5–LRRC58 complex with or without CDO1. Bottom, fractions across each peak were analysed by SDS–PAGE and Coomassie staining. SEC was repeated three times with similar results. **i**, TR-FRET assessment of complex formation between CDO1 and eGFP–LRRC58–ELOB–ELOC (top) and displacement of eGFP–LRRC58 by unlabelled LRRC58–ELOB–ELOC (bottom). **j**, AlphaFold modelling of the cullin–RING E3–ligase complex involving RBX2, CUL5, ELOB, ELOC, LRRC58 and CDO1. ELOB/C, ELOB–ELOC complex. Created in BioRender. Xiao, H. (2025) https://BioRender.com/ajl3ub6. **k**, Predicted aligned error plot of the CDO1–CRL5–LRRC58 interfaces. **l**, Time course of ubiquitylation of CDO1 by CRL5–LRRC58 with all reaction components (ATP, UBA1, UBE2D3, CRL5–LRRC58 and CDO1). Experiments were repeated three times with similar results. **m**, Ubiquitylation of CDO1 by CRL5–LRRC58 at endpoint (10 min) with individual components removed. Experiments were repeated three times with similar results. Two-tailed Student’s *t*-test for pairwise comparisons (**a**–**f**). Data are mean ± s.e.m.

To examine the mechanism through which LRRC58 regulates hypotaurine and taurine production from cysteine, we performed proteomics in the context of LRRC58 depletion (Supplementary Table 7). We found that CDO1 protein abundance increased up to eightfold in LRRC58-knockdown (LRRC58^KD^) primary brown adipocytes, the largest change in abundance across the entire proteome (Fig. 3a,b). Similarly, CDO1 abundance was selectively increased up to sevenfold in LRRC58^KD^ primary hepatocytes (Fig. 3c,d). Conversely, LRRC58 overexpression lowered CDO1 protein abundance in Hep G2 cells by 60% (Fig. 3e,f). The increase in hypotaurine and taurine abundance upon LRRC58 knockdown was completely reversed by depletion of CDO1 (Extended Data Fig. 6l,m), indicating that the effect of LRRC58 on taurine metabolism occurred via modulation of CDO1 abundance.

To examine whether LRRC58 affects CDO1 abundance through direct interaction, we first re-analysed BioPlex 3.0, which comprises stringent affinity purification–MS (AP–MS) experiments of 10,128 human proteins^36^. This analysis indicated that LRRC58 was a high-confidence and selective physical interactor with CDO1 and CUL5 (Extended Data Fig. 6n,o). LRRC58 has been also found to interact with ELOB in Jurkat cells^47^. To complement these experiments, we overexpressed Flag–LRRC58 in Hep G2 cells and used it as bait for AP–MS, identifying ELOB and ELOC as the top interacting proteins (Extended Data Fig. 6p,q). ELOB, ELOC and CUL5 form a Cullin 5–RING E3 ubiquitin ligase (CRL5) complex that mediates protein degradation by ubiquitylation of substrate proteins^48^, and a substrate adaptor is required to mediate the interaction between this complex and its substrates^49^. These led us to hypothesize that LRRC58 may be an E3 substrate adaptor that forms a complex with CRL5, which mediates ubiquitylation and degradation of CDO1. Flag immunoprecipitation followed by western blotting supported the interaction of LRRC58 with CDO1, CUL5, ELOB and ELOC (Fig. 3g). To reconstitute these interactions in vitro, we recombinantly expressed and purified CRL5–LRRC58 (comprising LRRC58, ELOB, ELOC, CUL5 and RBX2) and CDO1, which upon co-incubation resulted in formation of a 202.936-kDA complex (Fig. 3h). Using a time-resolved Förster resonance energy transfer (TR-FRET) assay, we determined a dissociation constant (*K*_d_) of 4.67 μM for the interaction between eGFP-tagged LRRC58 and terbium-chelate-labelled CDO1 (CDO1^Tb^) (Fig. 3i). The specificity of this interaction was further demonstrated by competing out pre-formed eGFP–LRRC58–CDO1_Tb_ complex with unlabelled LRRC58 (Fig. 3i). These experiments confirmed that the CRL5–LRRC58 complex binds directly to CDO1 and that LRRC58 may thereby act as a substrate adaptor for the CRL5–LRRC58 ubiquitin ligase.

**Extended Data Fig. 7:**
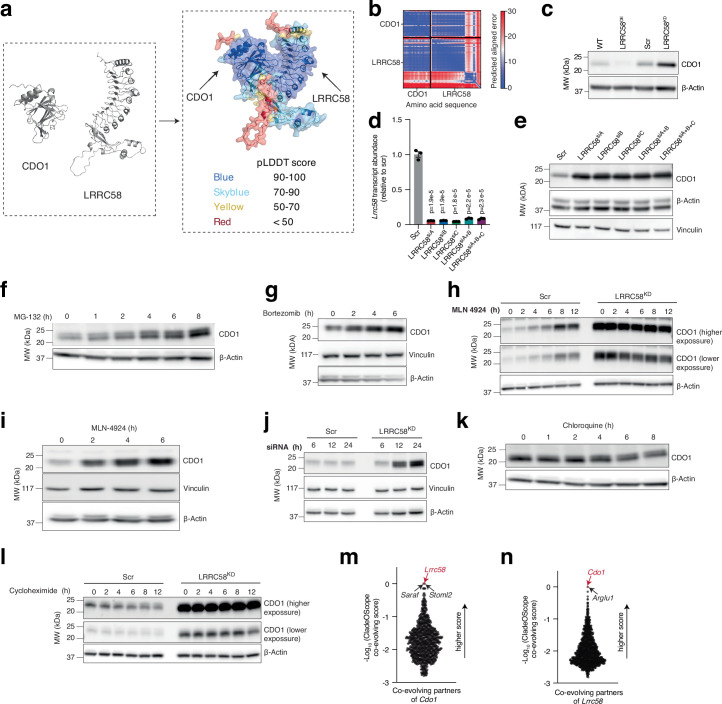
LRRC58 regulates CDO1 protein abundance. (**a**) Alphafold-Multimer modeling of direct physical interaction interface between CDO1 and LRRC58. The predicted local distance difference test (pLDDT) was used to examine the confidence of prediction. High pLDDT scores indicate high confidence of the amino acid residue structure. A score over 90 represents the highest confidence level; a score between 70–90 represents residues well-modeled with good backbone prediction; a score between 50–70 is of lower confidence and should be treated with caution; and score of lower than 50 indicates a low confidence prediction. (**b**) Predicted aligned error (PAE) plot of the interfaces between LRRC58 and CDO1. (**c**) CDO1 protein abundance in wildtype (WT), LRRC58 overexpression (LRRC58^OE^), scramble (scr), and LRRC58 knockdown (LRRC58^KD^) in Hep G2 cells. Representative blot from n = 3 cell replicate experiments shown. (**d**) Transcript abundance of *Lrrc58* in scramble (scr) compared to LRRC58 siRNA-treated primary hepatocytes for western blot analysis in panel **e**. n = 3 cell replicates. (**e**) Abundance of CDO1 protein in response to LRRC58 depletion in primary hepatocytes. Representative blot from n = 3 cell replicate experiments shown. (**f**) Time course of CDO1 protein abundance in response to inhibition of the proteasome with MG132 in primary hepatocytes. Representative blot from n = 3 cell replicate experiments shown. (**g**) Time course of CDO1 protein abundance in response to inhibition of the proteasome with Bortezomib in primary hepatocytes. Representative blot from n = 3 cell replicate experiments shown. (**h**) Time course of CDO1 protein abundance in response to NEDD8-activating enzyme (NAE) inhibition with MLN4924 in scramble (scr) and LRRC58 knockdown (LRRC58^KD^) primary hepatocytes. n = 2 cell replicates. (**i**) Time course of CDO1 protein abundance in response to MLN-4924 in primary hepatocytes. Representative blot from n = 3 cell replicate experiments shown. (**j**) Time course of CDO1 protein abundance in response to scramble (scr) and LRRC58 knockdown (LRRC58^KD^) primary hepatocytes. n = 2 cell replicates. (**k**) Time course of CDO1 protein abundance in response to inhibition of autophagy with chloroquine in primary hepatocytes. Representative blot from n = 3 cell replicate experiments shown. (**l**) Time course of CDO1 protein abundance in response to inhibition of protein synthesis with cycloheximide^91^ in scramble (scr) and LRRC58 knockdown (LRRC58^KD^) primary hepatocytes. n = 2 cell replicates. (**m**) Co-evolving partners of *Cdo1* gene based on clades^52^, which represent unbroken lines of evolutionary descent. A -log_10_ transformed CladeOScope score of 0 represents the top co-evolving gene partner. (**n**) Co-evolving partners of *Lrrc58* gene based on clades^52^, which represent unbroken lines of evolutionary descent. A -log_10_ transformed CladeOScope score of 0 represents the top co-evolving gene partner. (two-tailed Student’s t-test for pairwise comparisons in d). Data presented as mean ± s.e.m.

We next examined the structural basis for the physical interaction of LRRC58 with CDO1. We performed computational co-folding experiments using AlphaFold2 multimer^50^, which predicted the physical interface between CDO1 and LRRC58 with extremely high confidence (Extended Data Fig. 7a,b). Addition of CUL5, ELOB and ELOC further improved the predicted aligned error between LRRC58 and CDO1 (Fig. 3j,k). Of note, in this model the E3 activity and neddylation region of CUL5 was positioned proximal to CDO1, suggesting an orientation that is permissive for CDO1 ubiquitylation (Fig. 3j).

To test whether LRRC58 regulation of CDO1 was mediated through the ubiquitin–proteasome system, we began by comparing CDO1 abundance in wild-type, LRRC58-overexpressing (LRRC58^OE^), scramble short interfering RNA (siRNA)-treated (scr) and LRRC58^KD^ Hep G2 cells. As expected, LRRC58 overexpression led to depletion of CDO1 protein, whereas LRRC58 knockdown increased CDO1 abundance (Extended Data Fig. 7c). Depletion of LRRC58 in primary hepatocytes also increased CDO1 protein abundance (Extended Data Fig. 7d,e). Application of the proteasome inhibitors^51^ MG132 or bortezomib led to accumulation of CDO1 in primary hepatocytes (Extended Data Fig. 7f,g and Supplementary Table 8), confirming that CDO1 degradation was proteasome-dependent. Similarly, inhibition of neddylation by MLN4924 led to accumulation of CDO1 (Extended Data Fig. 7h,i and Supplementary Table 8), and this effect was lost upon LRRC58 knockdown (Extended Data Fig. 7h). We examined the kinetics of loss of LRRC58 on CDO1 abundance and observed CDO1 stabilization within hours of depletion of LRRC58 (Extended Data Fig. 7j and Supplementary Table 8). By contrast, inhibition of autophagy did not prevent CDO1 degradation (Extended Data Fig. 7k and Supplementary Table 8). To further examine whether CDO1 degradation is dependent on LRRC58, we blocked protein synthesis and profiled CDO1 protein half-life as a function of LRRC58 abundance. In this context, we found that CDO1 was stabilized in LRRC58^KD^ cells compared with controls (Extended Data Fig. 7l and Supplementary Table 8). We next determined whether LRRC58 directly mediates CDO1 ubiquitylation. We reconstituted CDO1, LRRC58 and interacting components of the E3 ligase (Fig. 3h) in an in vitro ubiquitylation assay, and found that CDO1 was ubiquitylated in an LRRC58-dependent manner (Fig. 3l,m).

These data, combined with AP–MS and structural modelling results, led us to conclude that LRRC58 is a substrate adaptor of an E3 ligase that targets CDO1 for ubiquitylation and degradation by the proteasome. Of note, phylogenetic profiling indicates that *Cdo1* and *Lrrc58* are conserved across evolution as highest-scoring co-evolving gene partners^52^ (Extended Data Fig. 7m,n). This suggests that LRRC58-mediated degradation of CDO1 has evolutionarily conserved biological roles. Supporting this notion, a study published during review of this work identified *Y42G9A.3* (also known as *Lrr2*), the *C. elegans* homologue of human *Lrrc58*, as a regulator of H_2_S production from cysteine that genetically relies on *Cdo-1* and modulates CDO1 protein levels^53^.

## LRRC58 complex responds to cysteine

**Fig. 4:**
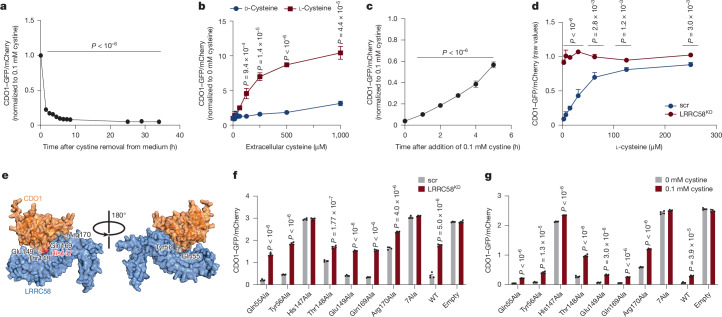
Regulation of LRRC58–CDO1 by cellular cysteine abundance. **a**, Post-translational stability of CDO1 reporter over time following switch from standard medium (0.1 mM cystine) to medium without cystine. Data are normalized to standard medium. *t* = 0, *n* = 10 cell replicates; other time points, *n* = 5 cell replicates. Statistical comparison is to *t* = 0. **b**, Post-translational stability of CDO1 following exposure to media with indicated levels of d-cysteine and l-cysteine for 24 h. Data are normalized to cells maintained in medium without cystine. No-cystine control, *n* = 8; other treatments, *n* = 4. Statistical comparison is between d-cysteine and l-cysteine. **c**, Reporter cells were cystine-depleted for 24 h, then changed back to normal 0.1 mM cystine medium for the indicated length of time. GFP/mCherry ratio is normalized to cells that were maintained in 0.1 mM cystine. *n* = 4 cell replicates. Statistical comparison is to *t* = 0. **d**, Concentration-dependent effect of cysteine on post-translational stability of CDO1 in LRRC58^KD^ or scr cells. *n* = 4 cell replicates. Statistical comparison is between LRRC58^KD^ and scr cells at each cysteine concentration. **e**, AlphaFold predicted structure of the interaction between LRRC58 and CDO1, with interface residues labelled. **f**, GFP/mCherry ratio in LRRC58^KD^ and scr Hep G2 cells expressing CDO1 mutant reporters. *n* = 4 cell replicates. **g**, GFP/mCherry ratio in Hep G2 cells expressing CDO1 mutant reporters treated with 0.1 mM cystine or cystine-restricted. *n* = 4 cell replicates. Two-tailed Student’s *t*-test for pairwise comparisons. Data are mean ± s.d.

**Extended Data Fig. 8:**
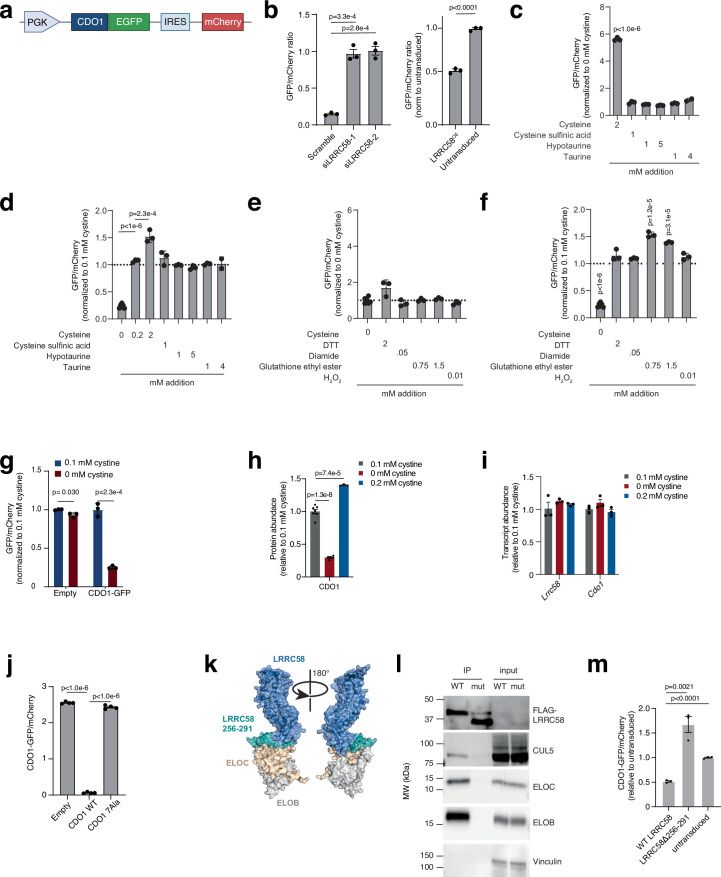
Cysteine is a signal regulating LRRC58-mediated CDO1 degradation. (**a**) Schema of CDO1 post-translational stability reporter including PGK promoter, CDO1-GFP fusion, internal ribosome entry sequence (IRES) and mCherry for transcriptional normalization. (**b**) Post-translational stability of CDO1 reporter in Hep G2 cells following transfection with siRNA targeting LRRC58 or scramble control (left) or overexpression of LRRC58 (right). n = 3 cell replicates. (**c**,**e**) Post-translational stability of CDO1 reporter in Hep G2 cells cultured in media without cystine and with the following compounds added for 26 h. n = 3 cell replicates except n = 6 for 0 mM cystine. Data are normalized to media without cystine. **(d**,**f**) Post-translational stability of CDO1 reporter in Hep G2 cells cultured in media with 0.1 mM cystine (equivalent to 0.2 mM cysteine; except the positive control without cystine) and with the following compounds added for 26 h. n = 6 for 0 and 0.2 mM cystine and n = 3 for other treatments. Data are normalized to 0.1 mM cystine media without further treatment. (**g**) Post-translational stability of the CDO1-GFP reporter or an empty vector (GFP-only) reporter after exposure to media with 0.1 mM cystine supplementation (equivalent to 0.2 mM cysteine), or media without cystine for 24 h. Data for each reporter are normalized to the 0.1 mM condition. n = 3 cell replicates. (**h**) Comparison of CDO1 abundance in primary hepatocytes cultured under cystine control (0.1 mM, equivalent to 0.2 mM cysteine), 0 mM cystine, or cystine supplementation (0.2 mM, equivalent to 0.4 mM cysteine). n = 7 (Control), n = 5 (0 mM cystine), and n = 3 (0.2 mM cystine) cell replicates. (**i**) Transcript abundance of *Lrrc58* and *Cdo1* in primary hepatocytes cultured under cystine control (0.1 mM, equivalent to 0.2 mM cysteine), 0 mM cystine, or cystine supplementation (0.2 mM, equivalent to 0.4 mM cysteine,). n = 3 (Control), n = 3 (0 mM cystine), and n = 3 (0.2 mM cystine) cell replicates. (**j**) CDO1-GFP/mCherry ratio of WT and 7Ala-CDO1 GFP fusions in media lacking cystine. n = 4 cell replicates. (**k**) Alphafold predicted structure of interaction between LRRC58 (blue), ELOB (white), and ELOC (tan). The putative ELOB-binding domain containing residues 256–291 of LRRC58 is teal. (**l**) Co-IP of FLAG-LRRC58 (WT) and FLAG-LRRC58∆256-291 expressed in Hep G2 cells. Immunoblots of CUL5, ELOB, ELOC, FLAG and vinculin are shown for the IP and input. Results are representative of 3 independent experiments. (**m**) CDO1-GFP/mCherry ratio of LRRC58WT and LRRC58∆256-291 expressing Hep G2 cells under cysteine restriction. n = 4 cell replicates. Statistics relative to untransduced. (two-tailed Student’s t-test for pairwise comparisons in b-j. b (left), c-h, and j were corrected with Bonferroni Dunn test). Data presented as mean ± s.e.m.

We next sought to understand the cellular signals that regulate LRRC58-mediated degradation of CDO1. We generated a fluorescent reporter of CDO1 post-translational stability (Extended Data Fig. 8a). This reporter recapitulated modulation of post-translational CDO1 stability upon LRRC58 knockdown and LRRC58 overexpression (Extended Data Fig. 8b). Using this system, we screened for factors that could regulate LRRC58-mediated CDO1 degradation. CDO1 determines conversion of cellular cysteine to taurine at the expense of cysteine contribution to other cellular processes such as glutathione production. Therefore, we reasoned that LRRC58-mediated degradation of CDO1 may be responsive to the abundance of metabolites in this pathway. We manipulated the abundance of major metabolites that are central to cysteine and taurine metabolism in the cell (Extended Data Fig. 8c,d). In addition, because cysteine regulates thiol redox homeostasis by regulating glutathione production, we additionally tested redox-active metabolites related to glutathione (Extended Data Fig. 8e,f). Remarkably, we observed robust regulation of post-translational CDO1 stability only upon modulation of cellular cysteine (Fig. 4a,b and Extended Data Fig. 8c–g). Cysteine depletion led to a rapid decrease of CDO1 post-translational stability (Fig. 4a), whereas increases in cysteine concentration led to post-translational stabilization of CDO1 (Fig. 4b,c). Notably, the effects of cysteine on post-translational CDO1 stability were dependent on LRRC58 (Fig. 4d). Regulation of CDO1 abundance was quantitative across the physiologic concentration range of cysteine (Fig. 4b) and specific to the l-cysteine enantiomer (Fig. 4b). We observed similar regulation of endogenous CDO1 in primary hepatocytes, whereby depletion of cysteine decreased CDO1 protein abundance, whereas supplementing cysteine completely reversed this effect (Extended Data Fig. 8h). Regulation of CDO1 protein abundance by cysteine was independent of effects on LRRC58 or CDO1 gene expression, indicating regulation of post-translational stability of CDO1 protein (Extended Data Fig. 8i).

We next examined the residues on CDO1 that are required for LRRC58 binding and CDO1 degradation. The modelled interaction interface showed seven CDO1 residues that directly contact LRRC58 and could comprise the degron (Fig. 4e). We generated a CDO1 reporter with mutations to alanine at all these positions (CDO1(7Ala)). In contrast to the wild-type reporter, CDO1(7Ala) did not show an increase in post-translational stability with LRRC58 knockdown (Fig. 4f) or a decline in post-translational stability upon cysteine depletion (Fig. 4g and Extended Data Fig. 8j). Next, we modified each position individually to alanine, which showed that mutation in a single locus (His147Ala) also led to complete resistance to LRRC58-dependent degradation (Fig. 4f). Moreover, CDO1(His147Ala) largely lost responsiveness to cysteine depletion (Fig. 4g). Thr148Ala and Arg170Ala mutations individually resulted in partial resistance to LRRC58-mediated degradation, but the combined mutations were not additive beyond the effect of His147Ala alone (Fig. 4f). On the basis of these findings, we conclude that His147 on CDO1 is required for the response to depletion of cellular cysteine through LRRC58-mediated degradation.

Additionally, based on the modelled interface, we deleted the putative ELOB-binding region of LRRC58 (residues 256–291) (Extended Data Fig. 8k). LRRC58(∆256–291) did not interact with CUL5, ELOB or ELOC (Extended Data Fig. 8l). LRRC58(∆256–291) increased the post-translational stability of CDO1 compared with wild-type LRRC58, consistent with a direct interaction between CDO1 and LRRC58 and the requirement of LRRC58 interaction with ELOB and ELOC for CDO1 degradation (Extended Data Fig. 8m).

### LRRC58 regulates hepatic cholesterol

CDO1 controls the production of taurine, a metabolite that is central to cholesterol catabolism, by conjugating to bile acids, promoting their excretion from the liver^2,45^. Excretion of bile acids also promotes excretion of free cholesterol from the liver^54^. Since CDO1 regulates a metabolic node that is central to liver cholesterol metabolism^43,55^, increasing CDO1 abundance and activity is a potential approach to lower liver cholesterol.

**Fig. 5:**
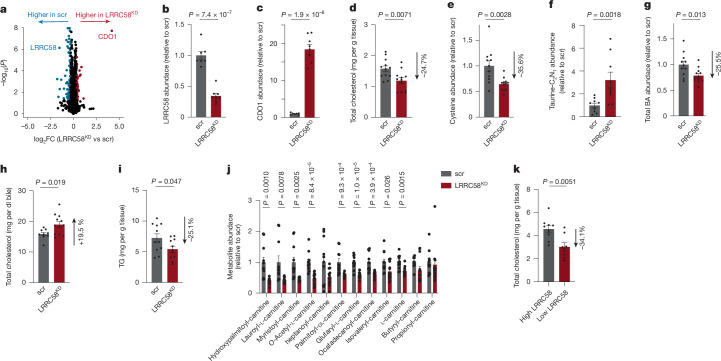
Depletion of LRRC58 stabilizes CDO1 and regulates hepatic cholesterol and fatty acid metabolism. **a**–**c**, Proteomics analysis (**a**) and LRRC58 (**b**) and CDO1 (**c**) abundance in the liver of WT and LRRC58^KD^ mice. Tissue specificity of LRRC58 knockdown is shown in Extended Data Fig. 9b,c. LRRC58^KD^, *n* = 8 male mice; scr, *n* = 7 male mice. Number of mice was limited by the throughput of a tandem mass tag (TMT) plex for proteomics. **d**, Hepatic cholesterol levels in WT and LRRC58^KD^ mice. LRRC58^KD^, *n* = 12 male mice; scr, *n* = 11 male mice. **e**, Hepatic cysteine levels in WT and LRRC58^KD^ mice. LRRC58^KD^, *n* = 12 male mice; scr, *n* = 11 male mice. **f**, ^13^C_2_^15^N_1_-taurine abundance in liver of scr and LRRC58^KD^ mice following intravenous administration of ^13^C_6_^15^N_2_-labelled l-cystine for 30 min. LRRC58^KD^, *n* = 9 male mice; scr, *n* = 9 male mice. **g**, Hepatic total bile acid (BA) levels in WT and LRRC58^KD^ mice. LRRC58^KD^, *n* = 12 male mice; scr, *n* = 11 male mice. **h**, Biliary cholesterol levels in WT and LRRC58^KD^ mice. LRRC58^KD^, *n* = 12 male mice; scr, *n* = 9 male mice (gallbladder extraction failed for 2 mice). **i**, Hepatic triacylglyceride (TG) levels in WT and LRRC58^KD^ mice. LRRC58^KD^, *n* = 11 male mice; scr, *n* = 11 male mice. **j**, Hepatic abundance of fatty acyl-carnitines in WT and LRRC58^KD^ mice. LRRC58^KD^, *n* = 12 male mice; scr, *n* = 11 male mice. **k**, Hepatic cholesterol levels measured in DO mice livers with highest (top 8%) LRRC58 abundance compared with lowest (bottom 8%) LRRC58 abundance. Two-tailed Student’s *t*-test for pairwise comparisons (**a**–**k**). Data are mean ± s.e.m. Source Data

**Extended Data Fig. 9:**
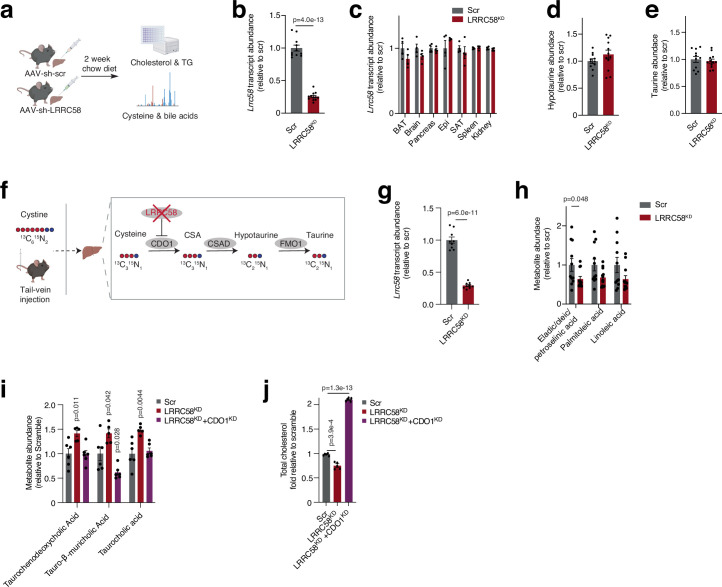
Proteomic and metabolomic changes in response to LRRC58 depletion in mouse liver. (**a**) Scheme for investigating liver cysteine, cholesterol, total triglycerides (TG), and bile acid levels in response to liver-specific LRRC58^KD^ in vivo. Created in BioRender. Xiao, H. (2025) https://BioRender.com/zub3s1i. (**b**) Transcript abundance of *LRRC58* in scramble (scr) compared to LRRC58^KD^ in the liver. n = 11 male mice, LRRC58^KD^ n = 12 male mice. (**c**) Transcript abundance of *LRRC58* in scramble (scr) compared to LRRC58^KD^ in other tissues. n = 4 male mice. (**d**, **e**) Hepatic taurine and hypotaurine abundance in scramble (scr) compared to LRRC58^KD^ mice. Scramble (scr) n = 11 male mice, LRRC58^KD^ n = 12 male mice. (**f**) Schema of stable isotope tracing using labeled ^13^C_6_^15^N_2_ -cystine to evaluate taurine flux in vivo following liver-specific LRRC58^KD^. Created in BioRender. Xiao, H. (2025) https://BioRender.com/1yuzp7v. (**g**) Transcript abundance of *Lrrc58* in scramble (scr) compared to LRRC58^KD^ in the liver of mice used for L-cystine (^13^C_6_^15^N_2_) tracing experiments. n = 9 male mice, LRRC58^KD^ n = 10 male mice. (**h**) Abundance of fatty acid species in the liver of scramble (scr) compared to LRRC58^KD^ mice. Scramble (scr) n = 11 male mice, LRRC58^KD^ n = 12 male mice. (**i**) Comparison of taurochenodeoxycholic acid, tauro-beta-muricholic acid, and taurocholic acid abundance in scramble (SCR) compared to LRRC58^KD^, and LRRC58^KD^ + CDO1^KD^ primary hepatocytes. n = 6 cell. (**j**) Cholesterol levels in scramble (scr) compared to LRRC58^KD^, and LRRC58^KD^ + CDO1^KD^ primary hepatocytes. n = 4 cell. (two-tailed Student’s t-test for pairwise comparisons in b-e & g-j). Data presented as mean ± s.e.m. Source Data

To test this, we performed adeno-associated virus (AAV)-mediated knockdown of LRRC58 in the liver of C57B/6J mice over the course of two weeks (Extended Data Fig. 9a–c). LRRC58 knockdown in liver led to an 18-fold selective increase of CDO1 protein in the liver (Fig. 5a–c and Supplementary Table 9). LRRC58 depletion decreased hepatic total cholesterol by 24.7% (Fig. 5d). This coincided with a 35.6% decrease in hepatic cysteine levels (Fig. 5e), indicative of LRRC58 depletion leading to increased cysteine partitioning to taurine (as observed in hepatocytes in Fig. 2d,f,g), and suggesting a consequent promotion of hepatic cholesterol conversion to bile acids. Unlike our observation in cultured primary hepatocytes (Fig. 2d), LRRC58 depletion in vivo did not lead to stable accumulation of hypotaurine and taurine in liver (Extended Data Fig. 9d,e and Supplementary Table 9). This was not unexpected, as liver taurine is rapidly released into the circulation in vivo^56^. To examine this further, we traced intravenous ^13^C^15^N-cysteine into liver (Extended Data Fig. 9f). These data showed that depletion of LRRC58 (Extended Data Fig. 9g) drove increased flux from cysteine to taurine in vivo (Fig. 5f). Hepatic taurine can be converted to taurine-conjugated bile acids and a variety of related species that are exported from the liver, many of which have only recently been identified^57^. In agreement, we observed 25.5% reduction of liver bile acids upon LRRC58 knockdown (Fig. 5g), suggestive of increased bile acid excretion. Since bile acid excretion promotes mobilization of hepatic free cholesterol to the gall bladder^54^, we measured cholesterol levels in the gallbladder and found that free biliary cholesterol content increased by 19.5% (Fig. 5h).

**Extended Data Fig. 10:**
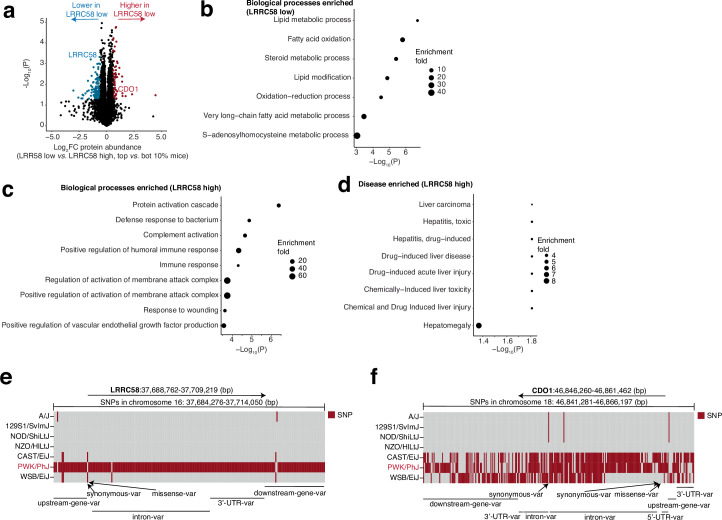
Proteomic and metabolomic consequences of natural variation in LRRC58 abundance in DO mice. (**a**) Liver protein abundance comparing DO mice with the bottom 10% LRRC58 abundance to those with top 10% LRRC58 abundance. n = 12 mice bottom 10%; n = 11 mice top 10%. (**b**) Gene ontology biological processes enriched in mice with bottom 10% LRRC58 abundance. (**c**) Gene ontology biological processes enriched in mice with top 10% LRRC58 abundance. (**d**) Disease networks enriched in mice with top 10% LRRC58 expression. (**e**) Single nucleotide polymorphisms of *Lrrc58* in DO founder strains with C57BL/6j as the reference. (**f**) Single nucleotide polymorphisms of *Cdo1* in DO founder strains with C57BL/6j as the reference. (two-tailed Student’s t-test for pairwise comparisons in a; one-sided Fisher’s exact test in b-d).

LRRC58 knockdown also lowered total triglycerides (TG), fatty acyl-carnitines, and fatty acid intermediates in the liver (Fig. 5i,j and Extended Data Fig. 9h), indicating a remodelling of hepatic fatty acid metabolism. These data are in line with previous reports identifying a role for CDO1 abundance in regulating fatty acid oxidation^58,59^. Importantly, modulation of hepatocyte cholesterol and taurine-conjugated bile acids upon LRRC58 knockdown was reversed by depletion of CDO1 protein (Extended Data Fig. 9i,j). These data indicated that LRRC58 effect on taurine metabolism occurred via modulation of CDO1 protein abundance. Finally, as our identification of LRRC58 was based on analysis of the DO cohort, we examined the role of natural genetic variation of LRRC58 on liver phenotypes (Extended Data Fig. 10a–f, Fig. 5k, and Supplementary Table 10), further discussed in Supplementary Discussion. Collectively, these data demonstrated that depletion of LRRC58 stabilized CDO1 in the liver, which was an effective approach to lower hepatic cholesterol and TGs.

## Discussion

Here we describe the development of MPCA, a resource that leverages covariation to systematically annotate functional relationships between metabolites and proteins. On this basis, we found that LRRC58 forms a basis for cellular sensing of cysteine and production of taurine, which is critical for cholesterol handling in the liver. It is logical that LRRC58-mediated degradation of CDO1 is responsive to cysteine, considering the position of CDO1 in cellular metabolism. When cysteine abundance is high, there is sufficient cysteine to fulfil its role in redox homeostasis by facilitating glutathione production and protein synthesis. Under these conditions, cysteine antagonizes LRRC58-mediated CDO1 degradation, allowing excess cysteine to shunt to taurine. When cysteine abundance is low, cysteine must be preserved for the essential functions of glutathione and protein synthesis. In this case, LRRC58-mediated degradation of CDO1 occurs to prevent catabolism of cysteine. CDO1 consumption of cysteine is a major mode of regulation of cellular cysteine levels^44^. Thus, it will be interesting to examine the role of LRRC58 in regulating cysteine metabolism in biological settings in which lowering cellular cysteine would be advantageous. These biological settings are widespread and include modulation of redox homeostasis^38^, iron metabolism^39^ and cysteine toxicity^40^. Moreover, it will be a clear priority to understand the mechanism through which LRRC58-mediated degradation of CDO1 is regulated by cysteine abundance.

Besides LRRC58, MPCA suggests many previously undescribed functional relationships between metabolites and proteins, which we provide to the research community with an interactive web interface (https://mpca-chouchani-lab.dfci.harvard.edu/). We envision that the annotations derived from the LASSO analysis can serve as a foundation for understanding protein–metabolite regulatory relationships.

## Methods

### Mice

A heterogeneous cohort of 163 female DO mice (24 weeks, *n* = 110; 18 months, *n* = 10; 22 months, *n* = 29; 28 months, *n* = 14) were used, and details were reported previously^10^. Mice were originally from the Jackson Laboratory^61^, derived from eight founder strains: A/J, C57BL/6J, 129S1/SvImJ, NOD/ShiLtJ, NZO/H1LtJ, CAST/EiJ, PWK/PhJ and WSB/EiJ. The cohort were group-housed in a temperature-controlled (20–22 °C) room on a 06:00 to 18:00 light:dark cycle upon arrival and fed a chow diet during acclimatization before transferring to a thermoneutrality incubator (29 °C) and fed a rodent high fat diet (OpenSource Diets, D12492) with 60% kcal% fat, 20% kcal% carbohydrate and 20% kcal% protein (ref. ^62^). Mice were fed ad libitum for eight weeks. All animal-related experiments were approved by Institutional Animal Care and Use Committee of the Beth Israel Deaconess Medical Center. Sample size for all animal work were chosen based on our previous work^10^. Randomization was performed to minimize batch effects. Researchers were not blinded to animal groups.

### Tissue extraction

Mice were euthanized by rapid cervical dislocation, and tissues were extracted and frozen in less than 20 s following euthanasia using the freeze-clamping method^63^. Freeze-clamped tissues were split into small pieces in liquid nitrogen and stored in a −80 freezer.

### Proteomics sample preparation

DO samples from each tissue were randomly assigned to 12 batches for TMT-based proteomics. Tissue pellets were weighed while frozen and lysed in the lysis buffer containing 100 mM 4-(2-hydroxyethyl)-1-piperazineethanesulfonic acid (HEPES) pH 8.5, 8 M urea, 2% sodium dodecyl sulfate (SDS) and one Roche cOmplete protease inhibitors tablet per 15 ml. Lysis was performed with Tissuelyser II (Qiagen) in a 4 °C cold room to an initial concentration of ~ 4–10 mg protein per ml buffer. After centrifugation, a bicinchoninic acid (BCA) assay was performed to measure protein concentration. On the basis of this measurement, samples were diluted to 1 mg protein per ml buffer. For each tissue, a ‘bridge’ sample was generated by mixing 50 μg of each sample, in order to serve as a standard to reflect the average abundance of each protein across the entire cohort. Two-hundred micrograms of protein from each sample were treated with 5 mM tris(2-carboxyethyl)phosphine (TCEP) at 37 °C for 1 h to reduce protein disulfide bonds, followed by addition of 25 mM iodoacetamide for 25 min at room temperature in the dark to alkylate free thiols. Methanol–chloroform precipitation^64^ was then performed to pellet proteins. The pellets were then resuspended in 200 mM *N*-(2-hydroxyethyl)piperazine-*N*′-(3-propanesulfonic acid) (EPPS) buffer pH 8, and digested with Lys-C and trypsin at an enzyme:protein ratio of 1:100 at 37 °C overnight. An additional round of 4 h trypsin digestion was performed the next day. The resulting mixture was then subjected to centrifugation and peptide quantification with microBCA (Thermo) kits. Based on this, 50 μg peptides from each sample were labelled by 100 μg of the TMTpro-16 reagents^65^ for 1 h at room temperature following the streamlined-TMT protocol^66^. Each TMT plex contained 15 randomized samples and a bridge sample. After a ratio check to confirm peptide loading in each TMT channel and TMT labelling efficiency, the reaction was quenched using 5 μl of 5% hydroxylamine for 15 min. All samples in a plex were then mixed based on the ratio check, desalted with Waters Sep-Pak cartridges, freeze-dried overnight using a speed-vac system. Three-hundred micrograms per TMT plex of dried peptides were resuspended in 10 mM ammonium bicarbonate pH 8.0, 5% acetonitrile (HPLC buffer A), and fractionated into 24 fractions with basic pH reversed-phase HPLC using an Agilent 300 extend C18 column. A 50-min linear gradient in 13–43% buffer B (10 mM ammonium bicarbonate, 90% acetonitrile, pH 8.0) at a flow rate of 0.25 ml min^−1^ was used for fractionation. Each fraction was then purified with StageTips, dried in a speed-vac, and reconstituted in a solution containing 5% acetonitrile (ACN) and 4% formic acid. Sample preparation for other proteomics experiments in this work was conducted using the same workflow as described above, without the bridge channel.

### LC–MS for proteomics

Two micrograms of peptides in each fraction were analysed by liquid chromatography–MS (LC–MS). Peptides were loaded onto a 100-μm capillary column packed in-house with 35 cm of Accucore 150 resin (2.6 μm,150 Å). Peptides were analysed by Orbitrap Eclipse Tribrid Mass Spectrometer (Thermo) coupled with an Easy-nLC 1200 (Thermo) using a 180-min gradient: 2%–23% ACN, 0.125% formic acid at 500 nl min^−1^ flow rate. A FAIMSPro^67^ (Thermo) device was used with compensation voltages at −40V/−60V/−80V. Data-dependent acquisition was used with a mass range of *m*/*z* 400–1,600 and 2 s cycles. MS1 resolution was set at 120,000, and singly charged ions were not further sequenced. MS2 was performed with standard automatic gain control (AGC) and 35% normalized collisional energy (NCE), and a dynamic exclusion window of 120 s and maximum ion injection time of 50 ms. Fragment ions were then selected for multi-notch SPS-MS3 method^68^ with 45% NCE to quantify TMT reporter ions. For AP–MS experiments, samples were analysed using the same LC–MS system. A 60-min gradient, with 2%–25% ACN and 0.125% formic acid was used for analysis without the FAIMSPro device. MS1 was performed with 120,000 resolution, 375–1,500 *m*/*z* scan range, and 50 ms maximum injection time. MS2 was performed with a 0.7 Th isolation window, 30% normalized NCE, 35 ms maximun ion injection time and 120 s window of dynamic exclusion. Only species with 2–5 charges were selected for analysis, and priority was set to species with lower charge states.

### Database searching

Database searching was conducted with the Comet algorithm^69^ on Masspike, an in-house search engine reported previously^17^. All mouse (*Mus musculus*) entries from UniProt (http://www.uniprot.org, downloaded 29 July 2020) and the reversed sequences, as well as common contaminants (for example, keratins and trypsin) were used for searching, with the following parameters: 25 ppm precursor mass tolerance; 1.0 Da product ion mass tolerance; tryptic digestion (cleaving at lysine and arginine residues) with up to three missed cleavages. Methionine artificial oxidation (+15.9949 Da) was set as a variable modification. Carboxyamidomethylation (+57.0215) on cysteine was set as a static modification. For TMT-based experiments, TMTpro (+304.2071 Da) on lysine and peptide N terminus were set as additional static modifications. Peptides were filtered with a target-decoy^17,70,71^ method to control the FDR to <1%. Parameters such as XCorr, ΔCn, missed cleavages, peptide length, charge state and precursor mass accuracy were used for filtering. Short peptides (<7 amino acids) were discarded. Proteins were assembled from peptides, and protein-level FDR was controlled, with the Picked FDR method^72^, to <1% combining all MS runs. For DO samples, an additional round of peptide filtering was applied, in order to remove peptides that are not shared across samples due to polymorphism^73^. All founder strain protein sequences (A/J, C57BL/6J, 129S1/SvImJ, NOD/ShiLtJ, NZO/H1LtJ, CAST/EiJ, PWK/PhJ and WSB/EiJ) were downloaded from Ensembl^74^ and in silico tryptic digested using the Protein Digestion Simulator (Pacific Northwest National Laboratory). A list containing peptides that are not shared across was used to filter out peptides from the DO experiments.

### Proteomics quantification

For TMT-based quantification, peptide abundance was measured by TMT reporter ions. Each reporter was scanned using a 0.003 Da window, selecting *m*/*z* with the highest intensity. Isotopic impurities were corrected based on the manufacturer’s specifications, and TMT signal-to-noise ratio (S/N) was used for quantification. Peptides with summed S/N lower than 320 across 16 channels of each TMT plex or isolation specificity lower than 70% were discarded. Proteins were quantified by summing up the TMT S/N values of peptides, and protein quantification was normalized to ensure equal protein loading across all TMT channels. For each DO sample, protein relative abundance was presented as log_2_ sample/bridge ratio, using the bridge sample in the same TMT plex as the biological sample. The values were analysed in both raw and median-centred formats^30,75^. Both returned nearly identical results for subsequent bioinformatic analyses, and we reported values without median-centring in this work. For AP–MS experiments, peptides were quantified based on peak area of MS1, and proteins were quantified by summing up the abundance of peptides.

### Metabolomics sample preparation

Metabolites from tissues were extracted with pre-chilled 80% methanol containing three internal standards (0.05 ng μl^−1^ thymine-d_4_, 0.05 ng μl^−1^ inosine-^15^N_4_, and 0.1 ng μl^−1^ glycocholate-d_4_), at a 4:1 buffer volume:sample mass ratio. Lysis was performed with Tissuelyser II (Qiagen) in a 4 °C cold room with three 30 s cycles using the highest power setting. Between every cycle, samples were chilled for 30 s. The mixture was spun in a 4 °C centrifuge at 18,000*g* for 15 min. The supernatant was collected for metabolomics analysis, and the pellet was saved for proteomics analysis. A ‘pool’ sample was generated by equally mixing all samples, representing the average metabolite abundance across all samples. Samples were then diluted tenfold with extraction buffer before loading to the LC–MS for analysis. For cell-based experiments, 12-well plates were used, and 100 μl of pre-chilled extraction buffer was used for every well. The mixture was then processed as described above.

### LC–MS and quantification for metabolomics

Ten microlitres of metabolite extracts were loaded onto a Luna-HILIC column (Phenomenex) using an UltiMate-3000 TPLRS LC with 10% buffer A (20 mM ammonium acetate and 20 mM ammonium hydroxide in water) and 90% buffer B (10 mM ammonium hydroxide in 75:25 v/v acetonitrile/methanol). A 10-min linear gradient to 99% mobile phase A was used to analyse metabolites. Liquid chromatography was coupled with a Q-Exactive HF-X mass spectrometer (Thermo). Liver metabolites were analysed by an LC–MS system consists of a Vanquish LC coupled with an Orbitrap Exploris 120 mass spectrometer (Thermo) using the same column and gradient. Negative or positive ion mode was used with full scan analysis over 70–750 *m*/*z* at 60,000 resolution, 10^6^ AGC, and 100 ms maximum ion accumulation time. In-source CID was applied at 5.0 eV. Ion spray voltage was 3.8 kV, capillary temperature was 350 °C, probe heater temperature was 320 °C, sheath gas flow was set at 50, auxiliary gas was set at 15, and S-lens RF level was set at 40. Metabolite peaks were analysed using TraceFinder (Thermo) software through a targeted approach. Peaks were matched to a metabolite library of ~800 validated metabolites on the LC–MS system, including metabolic tracers and peak area was used to quantify metabolite abundance. To account for run-to-run variations, metabolite abundance was adjusted by the average peak area of three internal standards. For bile acids, standards for individual bile acid species were used to acquire the retention time for subsequent quantification. Total bile acid content was obtained by summing up the peak areas of individual species. For DO samples, the sample loading sequence was randomized, and a pool was run every ten biological samples. This pool was used to calculate a sample-to-pool ratio for every metabolite in the ten sample runs before the pool, representing the relative abundance of a metabolite in a sample compared to the average abundance of this metabolite across the entire cohort. Other targeted LC–MS analyses of metabolite extracts were performed on a Vanquish HPLC System coupled to an Exploris 120 mass spectrometer equipped with a HESI ion source (Thermo Fisher Scientific). The LC system was controlled by Chromeleon 7.3.1 (Thermo Fisher Scientific), and the MS is controlled by Xcalibur 4.7.69.37 (Thermo Fisher Scientific). When analysing hypotaurine, taurine, cysteine and cystine, metabolites were separated on the Luna-HILIC column as described above. When analysing bile acid and bile acid-taurine conjugates, metabolites were separated on an ACQUITY UPLC HSS T3 column (150 mm × 2.1 mm, particle size 1.8 μm) maintained at 35 °C. Solvent A: 5% acetonitrile in water with 0.1% formic acid; solvent B: 5% water in acetonitrile with 0.1% formic acid. A flow rate of 0.4 ml min^−1^ was used and A/B gradient was as follows: being isocratic at 1% B for 5 min, linearly increasing to 99% B at 17.5 min, keeping at 99% B for 3.5 min, shifting back to 1% B in 0.1 min and holding at 1% B until 25 min. Mass spectrometer parameters: spray voltage positive electrospray ionization (ESI) mode +3.7 kV or negative ESI −2.5 kV; ion transfer tube temperature 275 °C; vaporizer temperature 320 °C; sheath gas 50 arbitrary units (a.u.); auxiliary gas 10 a.u.; sweep gas 1 a.u.; S-lens RF 70%; resolution 120,000; AGC target standard. The instrument was calibrated with FlexMix calibration solution (Thermo Fisher Scientific). When using Luna-HILIC column, each sample was analysed in ESI- mode; when using T3 column, each sample was analysed in ESI+ and ESI− switching mode. The *m*/*z* range was 70−800. Identity of metabolites had been confirmed with standards. Quantification of LC–MS data were performed through peak detection and integration functions in Freestyle software (Thermo Fisher Scientific) using mass ranges of calculated [M-H]^−^ *m*/*z* ± 5 ppm. In the case of quantifying stable isotope-incorporated metabolites, *m*/*z* window was manually examined to ensure exclusion of natural isotopes.

### Hepatocyte isolation and transfection

Primary hepatocytes were isolated from 8- to 10-week-old male C57BL/6 mice by liver perfusion. Livers were perfused with liver digest medium (Invitrogen, 17703-034). The cell suspensions were filtered through a 70-µm cell strainer. Primary hepatocytes were collected by a Percoll (Sigma, P7828) gradient centrifugation. Cells were cultured in Dulbecco’s Modified Eagle Medium (DMEM) with 25 mM glucose, 10% fetal bovine serum (FBS), 2 mM sodium pyruvate, 1% penicillin/streptomycin, 1 μM dexamethasone and 100 nM insulin. After 12 h, plating medium was removed and incubated with maintenance medium (DMEM with 25 mM glucose, 0.2% bovine serum albumin (BSA), 2 mM sodium pyruvate, 1% penicillin/streptomycin). Cells were collected within 48 h. For transient transfection, hepatocytes were transfected with LRRC58 siRNAs (Sigma Aldrich, SASI_Mm02_00347085 and SASI_Mm01_00138492) or CDO1 (Sigma Aldrich, SASI_Mm01_00121551) using RNAiMAX (Invitrogen). SASI_Mm01_00138492: sense strand 5′-GUAUGACCCUCCGACUCUU[dT][dT]-3′; antisense strand 5′-AAGAGUCGGAGGGUCAUAC[dT][dT]-3′. SASI_Mm02_00347085: sense strand 5′-CUCAGAAGAUGAAGCCAGU[dT][dT]-3′; antisense strand 5′-ACUGGCUUCAUCUUCUGAG[dT][dT]-3′. SASI_Mm01_00121551: sense strand 5′-GAAGUUUAAUCUGAUGAUU[dT][dT]-3′; antisense strand 5′-AAUCAUCAGAUUAAACUUC[dT][dT]-3′.

### Generation of LRRC58 overexpression Hep G2 line

Hep G2 cells were obtained from ATCC, authenticated by short tandem repeat profiling, and tested for mycoplasma contamination with mycoplasma-negative results. Cells were grown in EMEM (ATCC 30-2003), supplemented with 10% FBS (GeminiBio, 100–106) and 1% penicillin/streptomycin (Corning, 30-002-CI). Cells were detached using 0.25% trypsin (Gibco, 25200-056) and subcultured every 3–4 days. To generate the overexpression line, 12 μg of LRRC58 overexpression plasmid containing a Flag tag and an HA tag (BioPlex^36^), 9 μg of psPAX2 (Addgene #12260), 4.5 μg of pMD2.G (Addgene #12259) were co-transfected into Lenti-X 293T cells with Lipofectamine 3000 according to the manufacturer’s instructions. The viral supernatant was filtered through a 0.45-μm polyethersulfone membrane (PES) filter 48 h post-transfection, and then infected into Hep G2 wild-type cell line in the presence of 8 μg ml^−1^ Polybrene. Medium change was done 24 h post-transfection to complete medium. After incubation in complete medium for 24 h, cells were selected with 2 μg ml^−1^ puromycin. After 5 days, cells were validated for LRRC58 expression by qPCR and proteomics.

### Generation of LRRC58 knockdown in Hep G2 line

To generate LRRC58 knockdown cells, single guide RNA (sgRNA) oligonucleotide were designed as follows: forward: CACCGGCGCGCAGCTCTAAGAGCG; reverse: AAACCGCTCTTAGAGCTGCGCGCC. The following sgRNA oligonucleotides were used to generate the negative control cell line: forward: CACCGTTCGAAATGTCCGTTCGGT; reverse: AAACACCGAACGGACATTTCGAAC. DNA corresponding to sgRNAs was cloned into LentiCRISPRv2 (Addgene #52961). To generate the cell line, 12 μg of LentiCRISPRv2 containing LRRC58 sgRNA, 9 μg of psPAX2 (Addgene #12260), 4.5 μg of pMD2.G (Addgene #12259) were co-transfected into Lenti-X 293T cells with Lipofectamine 3000 according to the manufacturer’s instructions. The viral supernatant was filtered through a 0.45-μm PES filter 48 h post-transfection, and then infected into Hep G2 wild-type cell line in the presence of 8 μg ml^−1^ Polybrene. Medium change was done 24 h post-transfection to complete medium. After incubation in complete medium for 24 h, cells were selected with 1 μg ml^−1^ puromycin. After 5 days, cells were validated by qPCR and proteomics.

### Immunoprecipitation of LRRC58 for immunoprecipitation–western blotting

Hep G2 LRRC58^OE^ cells lysed using radioimmunoprecipitation (RIPA) lysis buffer (Thermo Fisher, 89900) and protease inhibitors (Roche, 11836153001). The cell lysates were centrifuged at 21,000 rpm for 10 min at 4 °C and the supernatants were used for subsequent analysis. Samples were immunoprecipitated overnight at 4 °C with anti-Flag (Sigma Aldrich, F1804; 4 µg of antibody per sample) that was coated on protein G magnetic beads (Invitrogen, 10007D). After incubation, the beads were washed 6 times with 0.5% NP-40 in phosphate buffered saline (PBS) and 4 times in PBS. Sample was eluted using SDS–PAGE reducing sample buffer and heat samples at 95°C in a heating block for 10 min.

### Immunoprecipitation of LRRC58 for AP–MS

Hep G2 LRRC58 overexpression cells were lysed in 0.5%. nonidet P-40 (NP-40) in PBS. The cell lysates were centrifuged at 21,000 rpm for 10 min at 4 °C and the supernatants were used for subsequent analysis. Samples were immunoprecipitated overnight at 4 °C with anti-Flag (Sigma Aldrich, F1804; 4 µg of antibody per sample) that was coated on protein G magnetic beads (Invitrogen, 10007D). A background control sample was generated by incubating the lysate with protein G magnetic beads that were not antibody-coated. After incubation, the beads were washed 5 times with 0.5% NP-40 in PBS and 5 times in PBS. Elution was achieved by 1% trifluoroacetic acid pH 2.5 at 60 °C for 5 min. Immunoprecipitated samples were dried in a speed-vac and then resuspended with 200 mM EPPS. Proteins were digested overnight at 37 °C with 4 μg trypsin and 4 μg Lys-C. Peptides were then purified by C18 stage tip and analysed by LC–MS as described above.

### Western blotting

Samples were isolated using radioimmunoprecipitation (RIPA) lysis buffer (Thermo Fisher, 89900) or IP lysis buffer (Pierce) with protease inhibitors (Roche, 11836153001). Homogenates were centrifuged at 21,000*g* for 10 min at 4 °C, and the supernatants were used for subsequent analyses. Protein concentration was determined using the BCA assay (Pierce). Protein lysates were denatured in Laemmli buffer (60 mM Tris, pH 6.8, 2% SDS, 10% glycerol, 0.05% bromophenol blue, 100 mM DTT), resolved by 4–12% NuPAGE Bis-Tris SDS–PAGE (Invitrogen), and transferred to a polyvinylidene difluoride (PVDF) membrane using an iBlot 2 (Invitrogen). Primary antibodies anti-β-actin (13E5, Cell Signaling Technologies, 1:1,000); anti-CDO1 (12589-1-AP, Proteintech, 1:1,000); anti-CUL5 (Bethyl Laboratories, A302-173A, 1:1,000), anti-ELOB (Proteintech, 10779-1-AP, 1:500), anti-ELOC (Proteintech 12450-1-AP, 1:1,000), anti-vinculin (Cell Signaling Technologies, 4650, 1:1,000), anti-Flag M2-HRP (A8592, Sigma Aldrich, 1:1,000) were diluted in tris buffered saline containing 0.05% Tween (TBS-T) and 5% BSA. Membranes were incubated overnight with primary antibodies at 4 °C. For secondary antibody incubation, HRP-conjugated secondary antibodies (Promega anti-mouse W402B, 1:5,000 or 1:10,000, and anti-rabbit W401B, 1:5,000 or 1:10,000) were diluted in TBS-T containing 5% BSA. Results were visualized with enhanced chemiluminescence (ECL) western blotting substrates (Pierce and ThermoScientific SuperSignal West Pico PLUS 34580).

### Immunoprecipitation of LRRC58 for Western blot

Flag–LRRC was immunoprecipitated from co-IP buffer with anti-Flag M2 magnetic agarose (Sigma Aldrich) and eluted with 3× Flag peptide (150 ng µl^−1^, Sigma Aldrich). Western blots were performed as above.

### Analysis of CDO1 degradation dynamics

Primary hepatocytes were plated at 2 × 10^5^ cells per ml into 6-well plates. Forty eight hours post-transfection cells were treated with cycloheximide for 0, 2, 4, 6, 8, 12 h at 20 μg ml^−1^. Abundance of CDO1 was measured by western blotting as described above.

### Generation of CDO1 post-translational stability reporter

Full-length CDO1 and CDO1 mutants were cloned into Cilantro 2 (gift from B. Ebert; Addgene plasmid #74450; http://n2t.net/addgene:74450; RRID:Addgene_74450) by Twist Bioscience. Lentivirus was packaged in Lenti-X 293T (Takara Bio) with pSpax2 and VSVG. Viral supernatant was filtered through a 0.45-µm filter. Hep G2 cells (ATCC) were spinfected in the presence of polybrene and selected in puromycin after 24 h. Empty Cilantro 2 vector was used as a negative control. After the indicated interventions, CDO1 post-translational stability was measured via flow cytometry on an LSR Fortessa flow cytometer (BD Biosciences) using the high-throughput sampler. Data were analysed with FlowJo v.10 by taking the ratio of the GFP mean fluorescence intensity to the mCherry mean fluorescence intensity in the mCherry^+^DAPI^−^ population.

### Flag–LRRC overexpression vector

Flag-LRRC and Flag-LRRC58^∆256–291^ were cloned by Twist Bioscience into pTwist Lenti CMV BSD.

### Media

Cystine depletion medium was generated from a base of Dulbecco’s MEM with l-glutamine and glucose without cysteine and methionine (US Biologicals, D9813, additional bicarbonate added at 1.5 g l^−1^) or Minimum Essential Medium Eagle without cystine, cysteine, glutamine or methionine (MP Biomedicals 091641454). As necessary, L-methionine (Sigma Aldrich M5308), l-glutamine (Sigma Aldrich G7513) and l-cystine dihydrochloride (Sigma Aldrich, C6727) were added to the final concentration used in EMEM (ATCC, 30-2003). Dialysed FBS was added at 10% (v/v)(Gibco) as well as 1% penicillin-streptomycin.

### siRNA in Hep G2 cells

Cells were reverse transfected with siRNA targeting LRRC58 or scramble with Lipofectamine RNAimax per the manufacturer protocol and assayed 48 h after transfection (Sigma Aldrich, SASI_Hs02_0032-1028 and SASI_Hs02_0032-1029 for LRRC58 and Mission Universal Negative Control #1 for scramble) using RNAiMAX (Invitrogen). SASI_Hs02_0032-1028: sense strand 5′-CAUUAAGAUUCGAAAUAUU[dT][dT]-3′; antisense strand 5′-AAUAUUUCGAAUCUUAAUG[dT][dT]-3′. SASI_Hs02_0032-1029: sense strand 5′-GAAAUCUGCCUUCUCUGAA[dT][dT]-3′; antisense strand 5′-UUCAGAGAAGGCAGAUUUC[dT][dT]-3′.

### Compounds used in reporter assays

Cells were treated with compounds including cycloheximide (Sigma Aldrich, 01810), l-cysteine (Thermo Scientific J63745.22), d-cysteine (Santa Cruz Biotechnology, sc-255054), MLN4924 (Selleck S7109), bortezomib (Selleck S1013), glutathione ethyl ester (Cayman Chemical 14953-50), diamide (Sigma Aldrich, D3648), taurine (Sigma Aldrich, T8691), hypotaurine (Sigma Aldrich H1384), hydrogen peroxide (Sigma Life Science, H1009), cysteine sulfinic acid (Sigma Aldrich, C4418) and dithiothreitol (Thermo Fisher, A39255).

### Inhibition of proteasome and autophagy

Primary hepatocytes were plated 2 × 10^5^ cells per ml into 6-well plates, then cells were treated with 10 μM MG132 for 0, 1, 2, 4, 6 or 8 h, or 30 μM chloroquine for 0, 1, 2, 4, 6 or 8 h. Abundance of CDO1 was measured by western blotting as described above.

### Inhibition of neddylation

Primary hepatocytes were plated at 2 × 10^5^ cells per ml into 6-well plates and treated with 10 μM MLN4924 for 0, 2, 4, and 6 h. Abundance of CDO1 was measured by Western blotting as described above.

### Liver LRRC58 knockdown in vivo

Male mice of C57BL/6J background were purchased from the Jackson Laboratory at 11 weeks of age and allowed 1 additional week for acclimatization feeding standard chow diet. AAVs (serotype 8) that carry short hairpin RNA (shRNA) targeting LRRC58 and AAVs that carry scramble shRNA were prepared by VectorBuilder (VB230509-1233rsh: TTAGCTGCAAGGACCATTAAG and VB010000-0023jze: CCTAAGGTTAAGTCGCCCTCG, respectively). At 12 weeks, a dose of 1 × 10^11^ genome copies per ml of AAV was administered as a bolus over 20 s by tail vein injection. Both scramble and LRRC58^KD^ cohorts were chow-fed for two weeks and were then subjected to cholesterol and triglycerides measurements, as well as metabolomics and proteomics. Mice were housed in a temperature-controlled (23 °C) room on a 12-h light-dark cycle. All animal-related experiments were approved by Institutional Animal Care and Use Committee of the Beth Israel Deaconess Medical Center.

### Gene expression quantification by qPCR

Total mRNA was extracted from animal tissues and cultured cells using TRIzol (Invitrogen), purified with a PureLink RNA Mini Kit (Invitrogen) and quantified using a Nanodrop 2000 UV–visible spectrophotometer. cDNA was prepared using 2 μg total RNA by reverse transcription–PCR (RT–PCR) using a high-capacity cDNA reverse transcription kit (Applied Biosystems), according to the manufacturer’s instructions. Real-time quantitative PCR (qPCR) was performed on cDNA using SYBR Green probes. qPCR was performed on a 7900 HT Fast Real-Time PCR System (Applied Biosystems) using GoTaq qPCR Master Mix (Promega). Reactions were performed in a 384-well format using an ABI PRISM 7900HT real-time PCR system (Applied Biosystems). Fold changes in expression were calculated by the ΔΔ*C*_t_ method using mouse RPLP0 as an endogenous control for mRNA expression. All fold changes are expressed normalized to the vehicle control. SYBR primer pair sequences were as follows. RPLP0: forward, 5′-AGATTCGGGATATGCTGTTGGC-3′; reverse,5′-TCGGGTCCTAGACCAGTGTTC-3′; LRRC58:forward, 5′- CGC GCC CTT CAG ACC C -3′: reverse, 5′-AGG TAT AAA CAT TCT AAA CTC CGC A-3′; CDO1: forward, 5′-GGGGACGAAGTCAACGTGG-3′; reverse, 5′-ACCCCAGCACAGAATCATCAG-3′.

### Cholesterol measurements

Total cholesterol was measured by Infinity Cholesterol assay (Thermo Scientific, TR13421). Approximately 50 mg liver was homogenized in PBS to a concentration of 200 mg ml^−1^ liver homogenate. Twenty microlitres of each homogenate was plated in 96-well clear-bottom UV plates (Thermo Scientific, 8404) and incubated at 37 °C in the presence of 20 μl of 0.25% deoxycholate for 5 min. Then, 200 μl of Infinity Cholesterol was added, followed by 15 min incubation at 37 °C. Absorbance was measured at 500 nm using a standard plate reader. Control Serum Wako I (Wako, 466-26201) was used in each assay as a standard for analysis of absorbances and quantification of cholesterol concentrations in Fig. 5d. Lipids were isolated from liver tissue utilizing 2:1 chloroform-methanol extraction. In brief, 20 µl of 2:1 chloroform-methanol per mg of liver tissue was used for homogenization followed by organic phase separation with 20% H_2_O (of the total volume) via centrifugation. The organic lipid-containing fraction was extracted, then vacuum dried and redissolved to a final concentration of 30 µl mg^−1^ in 1× reaction buffer. Total cholesterol was then determined using the Amplex Red Cholesterol Assay Kit (Thermo Fisher, A12216) following the manufacturer instructions and measured using a 96-well fluorescent plate reader (BMG Labtech, CLARIOstar) with excitation of 545 nm and emission at 590 nm in Fig. 5k.

### Total triglycerides level measurements

Triglycerides were measured by Infinity Triglycerides assay (Thermo Scientific, TR22421). Approximately 50 mg of liver was homogenized in PBS to a concentration of 50 mg ml^−1^ liver homogenate. 20 μl of each homogenate was plated in 96-well clear-bottom UV plates (Thermo Scientific, 8404) and incubated at 37 °C in the presence of 20 μl of 1% deoxycholate for 5 min. Then 200 μl of Infinity Triglycerides was added followed by 15 min incubation at 37 °C. Absorbance was measured at 500 nm using a standard plate reader. Control Serum Wako I (Wako, 466-26201) was used in each assay as a standard for analysis of absorbances and quantification of triglyceride concentrations.

### Cholesterol measurements in cells

Cholesterol levels were measured using total cholesterol Assay Kit (Colorimetric, STA-384; Cell Biolabs) according to the manufacturer’s instructions. Cholesterol content was normalized to the cell number.

### Cystine tracing

Tracing medium was prepared from DMEM powder with l-glutamine, glucose, and without cysteine or methionine (US Biological Life Sciences, D9813). This medium was then supplemented with l-methionine (0.01500 g l^−1^), sodium bicarbonate (3.7 g l^−1^), 0.2% heat-shocked BSA and 2% penicillin-streptomycin. Primary hepatocytes were transfected with LRRC58 siRNA (Sigma Aldrich, SASI_Mm02_00347085) using RNAiMAX (Invitrogen) following the protocol supplied by the manufacturer. Primary hepatocytes were plated in 12-well plates and cultured with plating medium (DMEM with 25 mM glucose, 10% FBS, 2 mM sodium pyruvate, 1% penicillin/streptomycin, 1 μM dexamethasone, and 100 nM insulin). The following morning, hepatocytes were incubated with maintenance medium (DMEM with 5 mM glucose, 0.2% BSA, 2 mM sodium pyruvate, 1% penicillin/streptomycin). Forty eight hours after transfection, the medium was changed to tracing medium with 0.03120 g l^−1^ l-cystine (^13^C_6_^15^N_2_, Cambridge Isotope Laboratories, 1252803-65-8). Cells were incubated in the tracing medium for 30 min. Metabolites from each well of cells were then extracted on ice with 125 μl cold 80 % methanol containing three internal standards: 0.05 ng μl^−1^ thymine-d_4_, 0.05 ng μl^−1^ inosine-^15^N_4_ and 0.1 ng μl^−1^ glycocholate-d_4_. Extracts were then subjected to metabolomic analysis as described above.

### Cystine tracing in vivo

Male mice on a C57BL/6J background were purchased from the Jackson Laboratory at 11 weeks of age and allowed 1 additional week for acclimatization feeding standard chow diet. AAVs (serotype 8) that carry shRNA targeting LRRC58 and AAVs that carry scramble shRNA were prepared by VectorBuilder (VB230509-1233rsh and VB010000-0023jze, respectively). At 12 weeks, a dose of 1 × 10^11^ genome copies per ml of AAV was administered as a bolus over 20 s by tail vein injection. Both scramble and LRRC58^KD^ cohorts were chow-fed for 2 weeks. After injection, 1 mg of l-cystine (^13^C_6_^15^N_2_, Cambridge Isotope Laboratories, 1252803-65-8) was administered as a bolus over 20 s by tail vein injection for 30 min. Samples were subjected to metabolomics, and qPCR. Mice were housed in a temperature-controlled (23 °C) room on a 12-h light-dark cycle. All animal-related experiments were approved by Institutional Animal Care and Use Committee of the Beth Israel Deaconess Medical Center.

### Cystine depletion and supplementation in primary hepatocytes

Medium was prepared from DMEM powder with l-glutamine, glucose, and without cysteine or methionine (US Biological Life Sciences- D9813). This medium was then supplemented with l-methionine (0.01500 g l^−1^), sodium bicarbonate (3.7 g l^−1^), 0.2% heat-shocked BSA and 2% penicillin-streptomycin. Primary hepatocytes were plated in 12-well plates and cultured with plating medium (DMEM with 25 mM glucose, 10% FBS, 2 mM sodium pyruvate, 1% penicillin/streptomycin, 1 μM dexamethasone, and 100 nM insulin). The following morning, hepatocytes were incubated with maintenance medium (DMEM with 5 mM glucose, 0.2% BSA, 2 mM sodium pyruvate, 1% penicillin/streptomycin). Twenty four hours after plating, the medium was changed to 0.03120 g l^−1^ l-cystine, no cystine or 0.0624 g l^−1^ l-cystine (Sigma Aldrich – C6727) overnight. Samples were extracted for proteomics and qPCR as described above.

### LRRC58 protein quantification via parallel reaction monitoring (PRM)

Proteins were extracted from frozen cell pellets using 200 μl of extraction buffer (100 mM Tris pH 8.5, 1% SDS, 1× Roche cOmplete Protease Inhibitor Tablet) with 2 μl of DNase 1 (Thermo Scientific) and were extracted at room temperature in a Thermo mixer at 900 rpm. Extracts were centrifuged at 13,000*g* and the supernatant was assayed for protein concentration using a BCA assay. Following quantification, 50 μg of each sample was reduced with 5 mM DTT for 30 min before alkylating with 20 mM IAM for 20 min. Samples were raised to 190 μl with water before adding 10 μl of 1:1 water-rinsed Sera-Mag carboxylated paramagnetic beads (Cytivia). Proteins were precipitated with 200 μl of 95 proof ethanol (Fisher Scientific) and incubated at room temperature for 5 min. The beads were washed twice with 80% ethanol, before resuspending in 75 μl of 100 mM EPPS (pH 8) with 1 μg of Trypsin (Fisher Scientific). Samples were incubated at 37 °C for 16 h, before clean up using a 50 mg C18 Sep-Pak (Waters), following manufacturer’s instructions.

Samples were reconstituted in 0.1% formic acid in water and three technical replicates of 600 ng were loaded onto Evosep tips per sample, following manufacturer’s instructions. The samples were analysed using an Evosep One: EV-1000 (Evosep) with the Evosep 15SPD method. The 15SPD method has a cycle time of 88 min where sample selection was performed in a randomized order. Detection of the peptides was performed on a timsTOF HT (Bruker) in prm-PASEF mode, with a mass range of 100–1,700 Da, ion mobility range of 0.60–1.6 V s cm^−2^, ramp rate of 9.42 Hz, and a ramp time of 100 ms. PRM targets were collected from 73 min to 83 min. The DLTYDPPTLLELAAR peptide from LRRC58 was triggered at an *m*/*z* of 844.4487 (+2 charge state) at an isolation width of 3 Da, between 0.90 V s cm^−2^ and 1.3 V s cm^−2^. Following acquisition, data were analysed in Compass Data Analysis (Bruker) using chromatogram integration at 844.4487 and 1.0995 V s cm^−2^.

### Constructs for biochemistry

For TR-FRET analysis: CDO1, LRRC58 and eGFP-LRRC58 were cloned into pAC-derived vectors^76^. CDO1 was expressed with an N-terminal StrepII–Avi tag, while LRRC58 and eGFP-LRRC58 with an N-terminal Flag tag. For SEC and in vitro ubiquitylation assays: LRRC58, CUL5, ELOB, ELOC and RBX2 were cloned into pAC-derived vectors. LRRC58 was expressed with an N-terminal StrepII tag, while RBX2 with an N-terminal Flag tag. CUL5, ELOB and ELOC were untagged. CDO1 was cloned into a pNIC-Bio2 derived vector with an N-terminal 6× His tag.

### Protein expression and purification

Baculovirus for protein expression of pAC-derived constructs were generated by transfection of expression plasmids into Sf9 cells at a density of 0.9 × 10^6^ cells per ml in ESF 921 medium (Expression Systems). Viral titre was increased by three rounds of infection in Sf9 cells. Strep–LRRC58, CUL5, ELOB, ELOC and Flag–RBX2 were co-expressed, while Strep-Avi-CDO1 was expressed separately. High Five cells were infected at a density of 1.8–2.0 × 10^6^ cells per ml in SF-4 Baculo Express ICM medium (BioConcept) and collected 40–48 h post-infection by centrifugation (1,500 rpm, 20 min). The collected cells were resuspended in lysis buffer (50 mM HEPES/NaOH pH 7.4, 200 mM NaCl, 0.1% (v/v) Triton X-100, 1 mM TCEP) supplemented with protease inhibitors and lysed by sonication, and the supernatant was treated with benzonase for 10 min. The cell lysate was clarified by ultracentrifugation (40,000 rpm, 1 h) and filtered. Filtered lysate was applied to Strep-Tactin XT 4Flow resin (IBA) equilibrated in lysis buffer. The resin was washed with wash buffer (50 mM HEPES/NaOH pH 7.4, 500 mM NaCl, 1 mM TCEP) and bound protein was eluted with elution buffer (50 mM HEPES/NaOH pH 7.4, 200 mM NaCl, 50 mM biotin, 1 mM TCEP). Further cleanup was done with anion exchange chromatography (buffer A: 50 mM HEPES/NaOH pH 7.4, 1 mM TCEP, buffer B: 50 mM HEPES/NaOH pH 7.4, 750 mM NaCl, 1 mM TCEP) followed by SEC (SEC buffer: 50 mM HEPES/NaOH pH 7.4, 150 mM NaCl, 1 mM TCEP). Flag–eGFP–LRRC58 and Flag–LRRC58 were each co-expressed with ELOB and ELOC in High Five cells and purified as described above with lysis and wash buffer instead containing 200 mM NaCl. Lysate was then applied to anti-Flag resin (Genscript) and eluted (50 mM HEPES/NaOH pH 7.4, 200 mM NaCl, 150 μg ml^−1^ Flag peptide) prior to ion exchange and SEC.

His–CDO1 was expressed in a LOBSTR *E.coli* expression strain (Kerafast). After transformation, 2 l cultures in TB medium were grown at 37 °C to an OD_600_ of ~0.6 and induced with 0.35 mM isopropyl β-d-1-thiogalactopyranoside (IPTG). Temperature was decreased to 18 °C, proteins were expressed overnight, and cultures were collected by centrifugation (3,300*g*, 20 min). Cell pellets were resuspended in His lysis buffer (50 mM HEPES/NaOH pH 7.4, 200 mM NaCl, 20 mM imidazole, 5% (v/v) glycerol, 1 mM TCEP) supplemented with protease inhibitors and lysed using sonication. After clearance by ultracentrifugation (39,000 rpm, 1 h), the supernatant was treated with benzonase for 10 min and incubated with high affinity Ni-charged resin (Genscript). Bound proteins were eluted with increasing imidazole concentrations (150–750 mM) and elution fractions were cleaned up by ion exchange chromatography (buffer A: 50 mM HEPES/NaOH pH 7.4, 2 mM TCEP, buffer B: 50 mM HEPES/NaOH pH 7.4, 750 mM NaCl, 2 mM TCEP) followed by SEC in ENL SEC buffer (30 mM HEPES/NaOH pH 7.4, 150 mM NaCl, 3 mM TCEP).

### Size-exclusion chromatography

Purified CRL5–LRRC58 (1.71 μM) was mixed with 2.57 μM CDO1 (a 1:1.5 molar ratio) in a final volume of 500 μl in SEC buffer (50 mM HEPES/NaOH pH 7.4, 150 mM NaCl, 1 mM TCEP). The mixture was incubated on ice for 1 h and then separated by SEC. CRL5–LRRC58 alone was separated by SEC to assess differences in co-elution of CRL5–LRRC58 with CDO1.

### TR-FRET assay

StrepII–Avi–CDO1 was biotinylated directly from elution fractions after affinity purification (which already contained 50 mM biotin) overnight in a 2 ml reaction composed of 5 mg of the eluted CDO1, 40 mM ATP, 10 MgCl_2_, and 300 μg BirA. Biotinylated CDO1 was then purified by SEC (SEC buffer: 50 mM HEPES/NaOH pH 7.4, 150 mM NaCl, 1 mM TCEP). To measure the *K*_d_ value of complex formation, 200 nM biotinylated CDO1 and 2 nM of Tb-coupled streptavidin were mixed in an assay buffer with 25 mM HEPES, 150 mM NaCl, 1 mM fresh TCEP, 0.5% BSA, and 0.05% Tween-20 (mixture 1). Serial dilutions of eGFP–LRRC58–ELOB–ELOC at a final concentration ranging from 9.8 nM to 20 μM were prepared in the same buffer (mixture 2). A total of 7.5 μl each of mixtures 1 and 2 were added to each well in a 384-well microplate and incubated for 1 h at room temperature and read on a Pherastar FS (BMG) plate reader with a 337/490/520 nm filter set. Background fluorescence due to eGFP–LRRC58–ELOB–ELOC was calculated by repeating the assay without CDO1 and subtracting the signal for the corresponding eGFP–LRRC58–ELOB–ELOC concentration. The ratio of emissions at 520/490 from five cycles were averaged as the final TR-FRET reading. *K*_d_ values were calculated using one-site specific binding and plotted in GraphPad Prism 10.

### Displacement assay

To confirm displacement of eGFP–LRRC58–ELOB–ELOC with unlabelled LRRC58–ELOB–ELOC, a final concentration of 200 nM biotinylated CDO1 and 2 nM of Tb-coupled streptavidin were mixed with 7 μM eGFP-LRRC58 in the assay buffer described above (mixture 3). Serial dilutions of unlabelled LRRC58–ELOB–ELOC at a final concentration ranging from 9.8 nM to 20 μM were prepared in the same buffer (mixture 4). A total of 7.5 μl each of mixtures 3 and 4 were added to each well in a 384-well microplate and incubated for 1 h at room temperature. Background fluorescence due to eGFP–LRRC58–ELOB–ELOC was calculated by repeating the assay without CDO1 and subtracting the signal for the corresponding eGFP–LRRC58–ELOB–ELOC concentration. The ratio of emissions at 520/490 from 5 cycles were averaged as the final TR-FRET reading. Half-maximal inhibitory concentration (IC_50_) values were calculated using an inhibitor versus response variable slope (four-parameter) model and plotted in GraphPad Prism 10.

### Ubiquitylation assay

To test ubiquitylation of CDO1 by CRL5–LRRC58, a 30 μl mixture of 1 μM CDO1, 0.5 μM CRL5–LRRC58, 0.2 μM of the E1 enzyme UBA1, 2 μM of the E2 enzyme UBE2D3, 5 mM ATP, 5 mM MgCl_2_, 25 mM HEPES/NaOH pH 7.5, 100 mM NaCl, 1 mM TCEP, 60 μM ubiquitin, and dH_2_O to bring the assay volume to 30 μl was prepared. A 6.5 μl aliquot of the reaction was taken and added to 13 μl of gel loading buffer as a time 0 timepoint prior to adding the ubiquitin to start the reaction. The reaction was incubated at 37 °C, and at time 2, 5, and 10 min, 7 μl of the reaction was taken and quenched in 13 μl gel loading buffer. Six microlitres from each timepoint was run on a 4–20% gel at 240 V for 22 min. For the control experiment, one component of the mixture was removed at a time and replaced with buffer (25 mM HEPES/NaOH pH 7.5, 150 mM NaCl, 1 mM TCEP) for a total of 6 reactions in a reaction volume of 10 μl, and reactions were quenched with 10 μl of gel loading buffer. For western blot analysis proteins were transferred onto PVDF membranes using an iBlot 2 dry blotting system (Thermo Fisher Scientific). A specific primary antibody was used to detect CDO1 (1:1,000 dilution anti-CDO1, Life Technologies 12589-1-AP). Blots were imaged using a LI-COR Odyssey CLx detecting an anti-rabbit secondary antibody (1:4,000 dilution Anti-Rabbit IgG, LI-COR, 92632211).

### Data analysis

All data analyses were performed using R 4.2.0 or Python unless otherwise noted. (1) *Assessment of TMT plex effect*. For each tissue type, 13 out of 163 samples were randomly selected and measured three times. Two times measured in the same TMT plex, and one time measured across 11 different TMT plexes. Principal component analysis (PCA) was used to examine clustering of samples on the basis of sample ID and measurement batch. (2) *Assessment of run-to-run variation of metabolomics*. Metabolomics samples were not multiplexed, therefore hundreds of LC–MS runs were required to complete metabolomic analysis of the DO samples. In order to assess the effect of the long running sequence on quantification of metabolites, four metabolic pool samples (representing the average abundance of metabolites across the entire DO cohort, see metabolomics sample preparation section above), were designated as ‘mock’ samples. These samples were treated as biological samples and spiked into the running sequence every ~50 runs. Since these mock samples were the same as pools, the theoretical sample-to-pool value of every metabolite in these samples should be 1. Batch effects were assessed by the deviation between the measured median values and 1. (3) *Assessment of protein contamination*. Proteins measured in DO samples were visualized in a PCA plot, and PC1 loading was extracted. Proteins with the top-2.5% PC1 rotation that deviated from the majority of the protein population were examined for tissue contamination using a well-established tissue-specific protein expression dataset^60^. Tissue-specific proteins in this dataset were identified using SILAC heavy-to-light ratio comparing the abundance of a protein in one tissue to its average abundance across all tissues. A total of 104 proteins that have muscle-specific expression, and with low expression in BAT were identified among proteins with top-2.5% PC1 loading as described above. Due to the nature of PCA analysis not allowing missing values, these proteins, along with proteins that had missing values and were highly correlated with these 104 proteins (Pearson’s *r* > 0.9) were removed from the BAT dataset, prior to downstream bioinformatic analysis. A total of 250 proteins were removed. (4) *Analysis of protein abundance*. Quantified proteins were mapped onto PaxDB^77^, a database for absolute protein abundance. Proteins were stratified based on expression levels represented in part per million (ppm) in the proteome. In each category, proteins quantified in this work were compared with those measured in the literature^12,21,22,78^. (5) *Analysis of proteomic and metabolomic variation*. Coefficient of variation (CV) was used as the measurement of proteomic and metabolomic variation, which is calculated by population standard deviation divided by population mean. CV of proteins and metabolites measured in this work were compared with previous works with deep omics coverage^27,29,63^. Quantification data of proteins and metabolites in this work were presented as log_2_ transformed values, and these values were transformed back before CV analysis. (6) *Metabolite annotation and ancestry analysis*. Every metabolite was first manually mapped on Chemical Entities of Biological Interest (ChEBI, https://www.ebi.ac.uk/chebi/), a database containing curated identifiers for chemical identities including metabolites. ChEBI IDs are used by major databases for metabolite identities, biochemical reactions and biological pathways. For every metabolite measured in MPCA, a list of ChEBI IDs were used to annotate the metabolite: the ChEBI IDs of the metabolite itself; the ChEBI IDs of its conjugate acids and/or bases; the ChEBI IDs of its salt adducts; the ChEBI IDs of the metabolite with different charge states; and ChEBI IDs of chemicals that have the same chemical formula and structure but named differently. Both primary and secondary ChEBI IDs were extracted. These IDs were then used for ancestry mapping using the ancestry mapping table provided by ChEBI. For instance, through ancestry mapping, ‘succinate’ was mapped onto ‘succinate^2-^’ (CHEBI:30031) then onto ‘dicarboxylic acid dianion’ (CHEBI:28965). This was to prepare for mapping MPCA protein–metabolite edges onto edges from established biochemical reactions (Rhea), pathways (Reactome) and transporters (TCDB), details described below. Without ancestry analysis, established edges between proteins and metabolites that are not at the bottom of the ChEBI hierarchy would become false negatives of the analysis. An example is RHEA-15421: a long-chain fatty acid + ATP + CoA = a long-chain fatty acid acyl CoA + AMP + diphosphate, enzyme: long-chain-fatty acid-CoA ligase 5 (ACSL5). Myristic acid is a long-chain fatty acid and a substrate to this reaction. The ‘ACSL5-a long-chain fatty acid’ edge was recapitulated by the ‘ACSL5-myristic acid’ edge in MPCA, through first mapping myristic acid (CHEBI:30807) to its ancestor long-chain fatty acid anion (CHEBI:57560). The top six levels of ChEBI hierarchy contain IDs with broad child chemical entities, and therefore were removed from ancestry mapping to prevent false positives from ambiguous edge mapping. (7) *Mapping MPCA edges onto established biochemical reactions, biological pathways and transporters of metabolites*. Established protein–metabolite relationships were downloaded from Rhea^32^ (https://www.rhea-db.org), Reactome^79^ (https://reactome.org) and TCDB^33^ (https://tcdb.org). Only edges with known evidence in mouse and human were used for this analysis. One node of the edge must be a protein, and the other node of the edge must be a metabolite. For every biochemical reaction, edges were generated between enzymes and substrates/products in this reaction. For pathways, direct protein–metabolite physical interactions and upstream and downstream regulation were used to establish edges. For transporters, edges were generated by connecting every protein transporter to all metabolites it transports. For each analysis, the list of edges was filtered to only contain proteins and metabolites measured in MPCA, ruling out edges not recapitulated due to measurement biases. For each tissue, a Fisher’s exact test was performed to examine the statistical significance of MPCA correlations recapitulating established protein–metabolite functional edges that reflect biochemical reactions, biological pathways, and transporters of metabolites. (8) *Visualization of protein–metabolite networks*. Protein–metabolite edges were visualized with Cytoscape v.3.9.1^80^. Every node was either a protein or a metabolite, and edges were only between a protein and a metabolite. To simplify the network, any protein–metabolite edge shared across multiple biochemical reactions were only counted once during network visualization. Edges from both BAT and liver were compiled in the same network. (9) *Analysis of features of MPCA-derived significant protein–metabolite correlations*. CVs for each metabolite were calculated using raw sample-to-bridge ratio without log_2_ transformation. These CVs were then correlated with the number of significant correlations for each metabolite. Number of total Rhea edges were correlated with the number of significant Rhea edges (Rhea edges that have been recapitulated by significant MPCA correlations) for each metabolite. Enrichment of protein population/classes among significantly correlating protein–metabolite pairs were performed using one-sided (right-sided) Fisher’s exact test. Protein populations were obtained from these databases: mitochondrial proteins: MitoCarta 3.0, mouse^81^; metabolic enzymes: mammalian metabolic enzymes database^82^; and kinases, Kinbase.com^83^. Gene Ontology (GO) term enrichment for protein correlates of each individual metabolite was performed using one-sided (right-sided) Fisher’s exact tests. GO: Biological Process and GO: Cellular Component datasets were obtained from https://www.ebi.ac.uk/QuickGO/. (10) *Linking accessory members to established metabolic pathways*. Significant pairwise protein–protein, protein–metabolite and metabolite-metabolite correlations were used in this analysis. The 1,411 Reactome lowest level mouse pathways were downloaded, and the size of each pathway ranged from 1 to 532 members. A Fisher’s exact test, adapted from our previous works^10,84^, was set up to calculate statistical enrichment of potential accessory proteins and metabolites associated with these established pathways. For a given established pathway, we tested its first-degree neighboring members for significant association with the pathway. For each individual test, we first counted the number of edges that linked the candidate member to the established pathway; then counted the number of edges the established pathway had to other proteins that were not the candidate member; then counted the number of edges the candidate member had to other members that were not a part of the established pathway; lastly counted edges that did not involve the established pathway nor the candidate member. These four numbers were used to set up the Fisher’s exact test. This test was looped through all established pathways. The resulting *P* values were subjected to multiple testing correction, separately in BAT and liver, using the Benjamini–Hochberg procedure^85^, and any association with an adjusted *P* value < 0.05 was considered significant. (11) *Enrichment analysis of MPCA pairwise correlations*. A protein–metabolite interaction network was constructed with nodes representing proteins and metabolites and edges denoting experimentally supported physical interactions. Protein–metabolite interactions were obtained from Rhea and TCDB, while protein–protein interactions were obtained from CORUM and BioPlex. For each protein–metabolite pair measured in MPCA, we computed the shortest path between the protein and metabolite within this combined network, the number of edges traversed in the path representing the hop distance between the species. Pairs with hop distance of one correspond to known physical interactions. Pairs separated by two or more hops were considered indirect functional associations. To assess the extent to which MPCA correlations are enriched for known interactions at each functional distance, we mapped MPCA protein–metabolite pairs to the reference network using ChEBI ancestry mapping, as described above, to account for cases where MPCA identifies chemically distinct species interacting with a protein. These mapped associations were then ranked by statistical significance (*P* value). For a given threshold *k*, we computed the fraction of the top-*k* MPCA associations that were found in the reference network at each hop distance. Enrichment was calculated by dividing the observed number of MPCA-discovered edges at a given distance in the top *k* by the number expected under random selection, given the total number of evaluable protein–metabolite pairs at that distance. This analysis was repeated across a range of *k* values to generate enrichment curves for each hop distance. The same analysis was also performed to evaluate the fold enrichment of LASSO associations that reached global statistical significance, FDR *q* < 0.05. (12) *Machine learning to discover protein regulators of metabolites*. Linear regression using the LASSO method^34^ was done using the R package glmnet. Relative protein abundance (log_2_ sample/bridge ratio) was used as the matrix to predict metabolite abundance (log_2_ sample/pool ratio). Data were not further scaled prior to analysis. A seed number of 100,000,000 was set to ensure reproducibility of the analysis. For all observations, the weight was set to 1. Data points were split to 90% and 10%, where for each iteration, 90% of the data were used for modelling, and 10% were used for validation. Cross-validation was performed using squared-error. Five values—0, 0.25, 0.5, 0.75 and 1—were set for mixing the relaxed fit with the regularized fit. To prevent overfitting, proteins with none-zero coefficients obtained at one standard error away from the minimum cross-validation error (lamda.1se, which gives the most regularized model) were used as the output. In addition, predicted metabolite abundance was correlated to the measured abundance to assess the modelling. (13) *Determination of FDR across all LASSO predictions*. To estimate statistical significance of LASSO-derived protein–metabolite associations, we performed ordinary least squares (OLS) regression for each protein using the metabolites with non-zero coefficients from that protein’s LASSO model. A two-sided *t*-test was then used to obtain *P* values. Resulting *P* values were subjected to Benjamini–Hochberg correction to control global FDR *q* < 0.05 was determined significant. (14) *Analysis of extreme outliers*. For each tissue, we rank-ordered the values of LASSO coefficients for all protein–metabolite LASSO predictions. Extreme outliers were determined based on IQR. Values lying beyond 3 times the IQR below Q1 or above Q3 were determined to be ‘extreme’ outliers (Q1 − 3 × IQR and Q3 + 3 × IQR). (15) *Assigning validation scores to metabolites*. Metabolites in each tissue were ranked based on the number of LASSO predictors with literature evidence in Rhea, Reactome and TCDB. To allow for comparison between tissues, a score ranged from 1–10 was given to each metabolite in each tissue by linearly scaling the number of its LASSO associations with literature evidence. The metabolite with the highest number of established LASSO edges was scored 10, while metabolites with non-zero predictors but no edge with literature evidence were scored 1. For every metabolite, its validation score in BAT and validation score in liver were then summed up for an overall validation score. (16) *Functional annotation of LASSO predictions*. LASSO hits for each metabolite were mapped onto CORUM and BioPlex to examine known protein interactors. If a newfound LASSO protein predictor of a metabolite physically interacts with a protein known to regulate this metabolite via a local metabolic network based on Rhea, TCDB and Reactome, then this LASSO hit was annotated as potentially regulating the metabolite through the known network. For instance, LRRC58 is found to physically interact with CDO1 based on BioPlex, and CDO1 and hypotaurine are known to be involved in R-MMU-1614558 and R-HSA-1614558, Degradation of cysteine and homocysteine (the hypotaurine-taurine pathway). Therefore, the newfound LRRC58-Hypotaurine edge is annotated as ‘May act through CDO1 and R-HSA-1614558 (Degradation of cysteine and homocysteine);R-MMU-1614558 (Degradation of cysteine and homocysteine)’. In addition, we annotated LASSO protein predictors of metabolites based on whether the corresponding protein is a known metabolic enzyme^82^, transporter based on TCDB^33^, or associated with mitochondrial function^81^. (17) *ROC and precision-recall analysis*. All correlations between proteins and metabolites in both liver and BAT were combined, and all non-zero LASSO coefficients identified in both tissues were merged to generate ROC and precision-recall curves. Threshold values for the curves were based on adjusted P values from pairwise correlation analysis, and FDR values from LASSO analysis. Analyses were done in Python. The ROC curves and area under the curve (AUC) were computed using the roc_curve and auc functions from scikit-learn, while the precision-recall curves and average precision were computed using precision_recall_curve and average_precision_score from the same library. The plots were visualized using Matplotlib and Seaborn. (18) *Re-analysis of BioPlex AP–MS data*. Unfiltered HEK 293T data were downloaded from https://BioPlex.hms.harvard.edu/interactions.php. Experiments with CDO1 and CUL5 as the bait proteins were extracted. CompPASS plus score and NWD score were calculated based on protein spectral counts in a given experiment and the frequency of this protein to appear in different AP–MS experiments, as detailed in BioPlex^36,84^. (19) *AlphaFold-Multimer modelling*. Three-dimensional structures of protein complexes were predicted using the ColabFold^86^ implementation of the AlphaFold-Multimer algorithm^87^. In each case, proteins were represented by their canonical sequences as reported by Uniprot. Five models were returned for each AlphaFold analysis, and the top model was selected based on ipTM Score. Each model was submitted multiple times, varying the order in which the constituent proteins were listed, and the highest-scoring model was retained. (20) *Co-evolution analysis*. This analysis was performed using CladeOScope (https://tabachlab.shinyapps.io/CladeOScope/), a web application for co-evolution analysis based on clades^52^, which represent unbroken lines of evolutionary descent. This analysis is typically used to uncover functional interactions between genes and proteins that have co-operative functions. The CladeOScope score of genes that co-evolved with *Lrrc58* or *Cdo1* were downloaded and −log_10_-transformed for plotting. A −log_10_-transformed CladeOScope score of 0 represents the top co-evolving gene partner. (21) *Analysis of enrichment of biological processes and disease networks in DO mice stratified by hepatic LRRC58 protein abundance*. Mice with liver LRRC58 abundance in the top-10% of our DO cohort were compared with those in the bottom 10%. Proteins with a fold change between these populations larger than 2 or smaller than 0.5 with two-tailed *t*-test *P* value smaller than 0.05 were defined as significantly upregulated or down regulated. Enriched biological processes were determined using the GOrilla GO analysis tool^88^. Significantly upregulated or downregulated proteins were used as the foreground, and all measured proteins in the liver were used as the background. Enrichment of disease networks were analysed based on protein involvement in disease networks downloaded from DisGeNET^89^. We filtered the list to only include liver diseases and network contained at least 3 members. For each disease network, enrichment was examined using Fisher’s exact test with upregulated or downregulated proteins as the foreground, and all measured proteins in the liver as the background and only enrichment but not depletion was considered. The resulting *P* values from all tests were then subjected to multiple test correction using the Benjamini–Hochberg procedure^85^, and any association with an adjusted *P* value < 0.05 was considered significant. (22) *Analysis of SNPs of* Lrrc58 *and* Cdo1. SNPs were found by comparing across all eight founder strains of the DO cohort, and C57BL/6J was set as the reference genome^61^. Based on the loci of the SNPs, potential consequences were assigned, such as upstream gene variant, missense variant or intron variant. SNPs with high confidence were included in this analysis, and data were downloaded from the Jackson Laboratory (https://churchilllab.jax.org/foundersnps/search#).

### MPCA web application

The MPCA web application (https://mpca-chouchani-lab.dfci.harvard.edu/) was hosted on the Shiny R server. The application was written and developed with the Shiny R package, and data visualizations were made possible with the following packages: shiny, tidyverse, ggpubr, visNetwork, png, dqshiny, DT, gsubfn, shinyjs, glue, shinydashboard and plotly.

### Statistical analyses

Quantification and statistical analysis pipelines are described in the sections above. Data analysis was performed in Excel, R, and GraphPad Prism. Data were expressed as mean ± s.e.m. unless otherwise noted, and *P* values were calculated using two-tailed Student’s *t*-test for pairwise comparison of variables. For enrichment analysis, Fisher’s exact tests were used. For correlation and enrichment analysis, *P* value was adjusted using the Benjamini–Hochberg procedure^85^, and adjusted *P* < 0.05 was considered significant. Sample sizes were determined based on prior reports using similar methods^10,12^. Unless otherwise stated, all stated replicates are biological replicates. For cell experiments, each biological replicate was originated from a shared genetically validated stock, independently plated, cultured for at least 48 h, and independently replated prior to the experiment. For MS analyses, sample order was randomized.

### Reporting summary

Further information on research design is available in the Nature Portfolio Reporting Summary linked to this article.

## Online content

Any methods, additional references, Nature Portfolio reporting summaries, source data, extended data, supplementary information, acknowledgements, peer review information; details of author contributions and competing interests; and statements of data and code availability are available at 10.1038/s41586-025-09535-5.

## Supplementary information

Supplementary Information Extended analysis of protein–metabolic pairwise correlations identified in MPCA.

Reporting Summary

Supplementary File 1 Uncropped western blots.

Supplementary Figure 1 Example flow cytometry gating.

Supplementary Tables Supplementary Tables 1–10.

## Source data

Source Data Fig. 5

Source Data Extended Data Fig. 9

## Data Availability

The mass spectrometry proteomics data have been deposited to the ProteomeXchange Consortium via the PRIDE^90^ partner repository with the dataset identifier PXD065355. Metabolomics data have been deposited to MassIVE under accession number MSV000098306. Other databases used in this work include UniProt (https://www.uniprot.org), Rhea (https://www.rhea-db.org), Reactome (https://reactome.org), TCDB (https://www.tcdb.org) and BioPlex (https://bioplex.hms.harvard.edu). Source data are provided with this paper.

## Code availability

R scripts for processing of proteomics and metabolomics data, pairwise correlation analysis, LASSO regression and website building have been deposited at Github (https://github.com/Xiaolab2024/MPCA).
