# Supplementary material for: Covariation MS uncovers a protein that controls cysteine catabolism
Source: Nature. 2025 Sep 17;647(8088):268–76. doi: 10.1038/s41586-025-09535-5 (PMC12589099; doi:10.1038/s41586-025-09535-5)
Supplement: Supplementary file 1 — Extended analysis of protein–metabolic pairwise correlations identified in MPCA. [file 41586_2025_9535_MOESM1_ESM.docx]

**Supplementary Text for “Covariation MS uncovers a protein that controls cysteine catabolism”**

Haopeng Xiao^1,2,3,4,*,#^, Martha Ordonez^1,2,*^, Emma C. Fink^1,2,5,*^, Taylor A. Covington^1,2^, Hilina B. Woldemichael^1,6^, Junyi Chen^3,4^, Mika Sarkin Jain^7,8^, Milan H. Rohatgi^7^, Shelley M. Wei^1^, Nils Burger^1,2^, Muneeb Sharif^1^, Julius Jan^3,4^, Yaoyu Wang^1^, Jonathan J. Petrocelli^1,2^, Katherine Blackmore^1,2^, Amanda L. Smythers^1,2^, Bingsen Zhang^1,2^, Matthew Gilbert^1^, Hakyung Cheong^1^, Sumeet A. Khetarpal^l^, Arianne Smith^1,2^, Dina Bogoslavski^1^, Yu Lei^1,2^, Laura Pontano Vaites^2^, Fiona E. McAllister^9^, Nick Van Bruggen^9^, Katherine A. Donovan^1,6^, Edward L. Huttlin^2^, Evanna L. Mills^10,11^, Eric S. Fischer^1,6^ & Edward T. Chouchani^1,2,12,#^

^1^Department of Cancer Biology, Dana–Farber Cancer Institute, Boston, MA, USA.

^2^Department of Cell Biology, Harvard Medical School, Boston, MA, USA.

^3^Department of Biochemistry, Stanford University School of Medicine, Stanford, CA, USA.

^4^Stanford Cancer Institute, Stanford University School of Medicine, Stanford, CA, USA.

^5^Department of Medical Oncology, Dana–Farber Cancer Institute, Boston, MA, USA.

^6^Department of Biological Chemistry and Molecular Pharmacology, Harvard Medical School, Boston, MA, USA.

^7^Department of Computer Science, Stanford University, Stanford, CA, USA.

^8^Harvard Medical School, Boston, MA, USA.

^9^Calico Life Sciences LLC, South San Francisco, CA 94080, USA.

^10^Department of Cancer Immunology and Virology, Dana-Farber Cancer Institute, Boston, MA, USA.

^11^Department of Immunology, Harvard Medical School, Boston, MA, USA.

^12^Howard Hughes Medical Institute, Chevy Chase, MD, USA.

* Co-first authors

# Corresponding authors: [hpxiao@stanford.edu](mailto:hpxiao@stanford.edu); [edwardt_chouchani@dfci.harvard.edu](mailto:edwardt_chouchani@dfci.harvard.edu)

In this **Supplementary Text** document, we extend our analysis of the MPCA resource.

**Additional analysis of pairwise metabolite enzyme substrate/product relationships.** Prominent examples of metabolite-protein relationships encompassing major aspects of cellular metabolism included NAD^+^ and succinate, which are metabolites involved in a variety of biochemical reactions recapitulated by MPCA. NAD^+^ is a major electron carrier involved in numerous redox reactions across the cell. MPCA determined NAD^+^ co-operativity with 81 NAD^+^-generating dehydrogenases including the major NAD^+^ producing enzyme complex in mitochondria, NADH dehydrogenase (Complex I) (**Figure 1d, e**). Similarly, succinate was found to correlate with succinate dehydrogenase (SDH) and 32 succinate-producing enzymes, such as prolyl hydroxylases Egl nine homolog 1 (EGLN1) and prolyl 3-hydroxylase 1 (P3H1) (**Figure 1d**). Notably, in the case of enzyme complexes comprising multiple subunits, such as SDH and Complex I, all mapped constituent subunits of the complex exhibited strong correlations with the corresponding metabolite pair **(Figure 1d, e)**. MPCA identified other well-known enzyme-substrate relationships across metabolite classes (**Extended data Fig. 1m, n**). For example, the amino acid deacetylation reaction catalyzed by aminoacylase 1 (ACY1): N-Acetyl-Methionine negatively correlated with ACY1, whereas the reaction product methionine positively correlated with the enzyme (**Extended data Fig. 1m**). Similarly, metabolites along the uridine monophosphate (UMP) catabolic pathway to uracil exhibited strong co-operativity directly upstream and downstream of uridine phosphorylase 1 (UPP1) (**Extended data Fig. 1n**). In total, significant protein-metabolite correlations in MPCA recapitulated 27% of all known molecular regulatory events derived from endogenous biochemical reactions mapped in our dataset (**Extended Data Fig. 1k, l**). Interestingly, as illustrated in the individual cases above, we observed variable directionality in enzyme-metabolite correlations observed in MPCA. The variable directionality of individual protein-substrate/product pairs across MPCA implicates underlying factors that contribute to individual enzyme-metabolite co-operativity, which is discussed in more detail in a later section.

**Additional analysis of pairwise relationships of metabolites and metabolite transporters.** MPCA captured established metabolite-protein relationships encompassing major aspects of cellular metabolism, including components of the mitochondrial electron transport chain (**Figure 1e**), amino acid metabolism (**Extended data Fig. 1m**), and nucleotide metabolism (**Extended data Fig. 1n**). These include well-established solute carrier (SLC) family proteins localized to the plasma membrane (PM), mitochondrial inner membrane (MIM), endoplasmic reticulum (ER) membrane, Golgi membrane, and vesicular membrane (VM) (**Extended Data Fig. 2d**). Transporters for norepinephrine^92,93^ and glutamate^94-98^ across different membranes were accurately recapitulated by MPCA. Likewise, SLC5A6 mediates the uptake of pantothenate and biotin across the plasma membrane^99^, which is reflected in a positive metabolite-transporter correlation by MPCA. Another example is the mitochondrial α-ketoglutarate/malate carrier SLC25A11, which exports malate from the mitochondrial matrix in exchange for α-ketoglutarate import^100^. MPCA derived co-operativity between SLC25A11 and both transport substrates (**Extended Data Fig. 2d**). We next examined substrate-transporter relationships not recapitulated by MPCA. We next examined substrate-transporter relationships not recapitulated by MPCA. We reasoned that this could be due to a known transporter not playing a determinative role in the steady-state abundance of a metabolite. For instance, glucose transporters SLC2A1, SLC2A3, and SLC2A4 were quantified in BAT. However, glucose abundance was not significantly correlated with these transporters. Instead, it correlated strongly with hexokinases, HK1, HK2, and HK3 (**Extended Data Fig. 2e**), which exert a high degree of flux control over glycolysis^101^. Similarly, ornithine was not significantly correlated with its known transporters quantified in the liver, SLC7A2 and SLC22A15. Instead, it correlated with the mitochondrial ornithine carbamoyltransferase OTC, and the mitochondrial ornithine aminotransferase OAT (**Extended Data Fig. 2f**). In addition, the tissue-specific nature of protein-metabolite co-regulation could exclude MPCA edges from recapitulating all possible metabolite-transporter relationships. Of the 482,043 significant protein-metabolite correlations identified, only 11,745 were shared between BAT and liver, highlighting tissue specificity of protein-metabolite relationships (**Extended Data Fig. 2g**). We additionally examined whether transporter redundancy would bias MPCA recapitulation towards metabolites with fewer transporters. However, this bias was not observed in MPCA (**Extended Data Fig. 2h**).

**Additional analysis of pathway level metabolite-protein co-operativity.** Continuing our analysis of MPCA derived pairwise correlations at the pathway level, we also analyzed protein classes that were enriched among metabolite-correlated proteins, and found a strong enrichment of metabolic enzymes, kinases, as well as mitochondrial proteins (**Extended Data Fig. 3c, d**). We next characterized directionality of correlation features and protein-metabolite co-operativity at the pathway level. To do so, we curated and filtered the Reactome^79^ database into minimal pathway modules representing 1,626 established coordinated metabolic pathways in human and mouse (**Extended Data Fig. 3e, f &** **Supplementary Table 3**). MPCA-derived covariation reconstructed 33% of known metabolic pathways assembled by molecules measured in MPCA **(Extended Data Fig. 3e, f)**.

It is noteworthy that MPCA recapitulation of established enzyme-substrate/product relationships could manifest as either positive correlation or negative correlation, depending on the protein-metabolite pair. Of the 1,373 RHEA enzyme-substrate/product relationships mapped in MPCA, 44% of known enzyme substrate/products exhibited positive abundance correlations with their corresponding enzyme, while 56% exhibited anti-correlations. Presumably, the directionality of these individual relationships is explained in part by factors that control rates of metabolic reactions in cells and tissues. For example, previous work indicates that in many cases metabolite concentrations are often the strongest drivers of net rates of cellular metabolic reactions at the pathway level^102^. Metabolic pathway enzymes calibrate through coordinated regulation of abundance^103,104^, and it would be expected that in these cases, steady state abundance of metabolic pathway intermediates would correlate positively with partner enzyme abundance. Conversely, in cases where an enzyme in a metabolic pathway exerts a high degree of flux control, one might instead expect an anti-correlation with the metabolite it consumes.

To explore these relationships further, we examined protein-metabolite co-operativity at the pathway level. (**Extended Data Fig. 3e-g & Supplementary Table 3**). Each pathway comprised a well-defined metabolic process, with up to 15 metabolite and 45 protein members per pathway, and MPCA-derived covariation reconstructed 33% of these pathways (**Extended Data Fig. 3f**). Prominent examples include the TCA cycle (**Extended Data Fig. 3h**) and glycolysis (**Extended Data Fig. 3i**). Interestingly, at enzyme nodes of metabolic pathways known to exert high degrees of flux control, MPCA tended to report negative correlations between the substrate metabolites and their corresponding enzyme. For example, α-ketoglutarate abundance correlated negatively with the abundance of the α-ketoglutarate dehydrogenase complex **(Extended Data Fig. 3h)**, while glucose abundance negatively correlated with hexokinases **(Extended Data Fig. 3i)**^103,105,106^. Notably, in these cases the downstream enzymes in the metabolic modules manifested similar correlations **(Extended Data Fig. 3h, i)**. Conversely, for intermediary pathway metabolites like succinate, co-operativity with SDH manifested as a positive correlation **(Extended Data Fig. 3h)**, supporting the notions of flux control described in the paragraph above. It is also worth noting that in the case of metabolic cycles like the TCA cycle, MPCA reported positive correlations with most intermediates (with the exception of substrates consumed by enzymes with more flux control like α-ketoglutarate^106^) **(Extended Data Fig 3h)**, further supporting the concept of metabolic enzyme calibration of metabolite utilization through coordinated regulation of abundance. Of note, an alternative possibility would be cases in which pathway flux is increased or maintained by an overall joint increase in pathway enzyme abundance while leading to lower concentrations of metabolites and negative association with its substrates and products. To examine this, we analyzed the 113 biochemical reactions where an enzyme and its substrate and product were both recapitulated by MPCA. Among these reactions, 40.71% had negative interactions between an enzyme and both its substrate and product, 38.94% had positive interactions for both, and 20.35% had one positive and one negative interaction.

**Accessory members of metabolic pathways.** We next examined whether correlations in MPCA could link accessory members to established metabolic pathways (**Extended Data Fig. 4a; Supplementary Table 4**). For each Reactome pathway, accessory members were scored by the enrichment of covariation edges between the pathway and a metabolite/protein that is not annotated as a part of the pathway (**Extended Data Fig. 4a**; see Methods)^10,84^. In BAT, 686 proteins and 62 metabolites were statistically significantly linked to 124 pathways (**Extended Data Fig. 4b**). In liver, 1,002 proteins and 218 metabolites were linked to 143 pathways (**Extended Data Fig. 4c**). Many plausible accessory members were linked to established metabolic pathways. For instance, dihydroxyfumaric acid associated significantly with the TCA cycle (**Extended Data Fig. 4d**). Dihydroxyfumarate has been proposed previously to enter the TCA cycle (R-MMU-71403) through an abiotic pathway^107^. Similarly, in the liver, oxidized glutathione (GSSG) and L-glutamine were found accessory to the glutathione synthesis and recycling pathway (R-MMU-174403) (**Extended Data Fig. 4e**). These examples suggest that MPCA correlations could reflect protein-metabolite relationships that are biologically meaningful but not already annotated in databases.

**Additional analysis of statistical properties of pairwise correlations in MPCA.** Here we examined the statistical properties of covariation derived from these different forms of metabolite-protein relationships. We rank ordered MPCA correlations by significance of correlation, and analyzed the fold enrichment over random selection using RHEA and TCDB as true positives for physical protein-metabolite interactions. We found that top-ranked correlations in MPCA annotated established direct physical interactions in RHEA and TCDB with higher fold enrichment compared to lower-ranked correlations **(Extended Data Fig. 4f)**. Intriguingly, we observed that this analysis biased towards metabolites that were well-characterized in the literature **(Extended Data Fig. 4g, h)**, such as adenosine-5’-diphosphate (ADP), NAD, and adenosine-5’-monophosphate (AMP), cofactors known to participate in many biochemical reactions. These well studied metabolites exhibited much higher fold enrichment than other metabolites in MPCA **(Extended Data Fig. 4h)**, suggesting our enrichment estimates were conservative, especially for MPCA-derived relationships involving understudied metabolites that are not well characterized in the databases. We also found that MPCA pairwise correlations preferentially recapitulated direct interactions over indirect associations (**SI Discussion & Extended Data Fig. 4i-l**). We then extended this analysis to examine whether MPCA pairwise correlations differentiate direct interactions from non-specific correlations induced by general metabolic processes. To do so, we constructed a metabolic interaction network incorporating experimentally validated metabolite-protein interactions from RHEA and TCDB, along with protein-protein interactions from CORUM and BioPlex (**Extended Data Fig. 4i**). Within this network, we defined direct interactions as known physical interactions reported in RHEA and TCDB, while indirect interactions were characterized by the number of intermediary steps ("hops") separating proteins and metabolites in the network (**Extended Data Fig. 4i, j**). Enrichment analysis comparing direct and indirect interactions revealed that MPCA pairwise correlations preferentially discovered direct interactions, with quantitatively declining enrichment for indirect associations as functional distance increases (**Extended Data Fig. 4k, l**).

We additionally analyzed precision-recall (PR) and receiver operating characteristic (ROC) curves using RHEA, TCDB, and Reactome as ground truth references to evaluate whether pairwise correlations in MPCA can be used to robustly uncover novel protein-metabolite relationships (**Extended Data Fig. 4m, n**). While a significant proportion of protein-metabolite relationships in these databases were recapitulated by MPCA (**Extended Data Fig. 1k, l, Extended Data Fig. 2b & Extended Data Fig. 3f, g**), a relatively low area under curve (AUC) and average precision (AP) of ROC/PR curves was observed (**Extended Data Fig. 4m, n**). We reason that this is in part because known protein-metabolite relationships are necessarily limited and biased towards well studied metabolites **(Extended Data Fig. 4g, h)**, leading to underestimation of true positives and overestimation of true negatives. In addition, the large number of significant pairwise correlations identified (**Supplementary Table 2**) necessitates a more stringent statistical approach for discovery work to prioritize proteins that play most dominant and specific roles in determining the abundance of individual metabolites, which is discussed in greater detail below.

**Additional analyses of LRRC58 in DO mice and liver metabolism.** We stratified the DO cohort by abundance of LRRC58 in the liver (**Extended Data Fig. 10a**) and analyzed enrichment of biological processes and disease networks as a function of LRRC58 abundance. We found that mice with low LRRC58 exhibited elevated CDO1 protein and proteins participating in lipid metabolic processes and fatty acid oxidation in the liver (**Extended Data Fig. 10a, b**), while those with high LRRC58 abundance had increased liver inflammatory markers (**Extended Data Fig. 10c**). Disease network analysis revealed enrichment of proteins implicated in promoting liver metabolic disease and cancer in mice with high LRRC58 abundance (**Extended Data Fig. 10d**). Moreover, DO mice with the highest LRRC58 protein expression also exhibited significant elevation in liver cholesterol compared to those with the lowest LRRC58 protein expression **(Figure 5k)**. In addition, we examined single nucleotide polymorphisms (SNPs) in *Lrrc58* and *Cdo1* genomic loci in the DO cohort. While potential coding variants were rare, we found 285 high-confidence potential regulatory SNPs for *Cdo1* and 234 for *Lrrc58*, with a high PWK founder strain effect on expression of both proteins (**Extended Data Fig. 10e, f and Supplementary Table 10**).

**SI References:**

10 Xiao, H. et al. Architecture of the outbred brown fat proteome defines regulators of metabolic physiology. Cell 185, 4654-4673 e4628, doi:10.1016/j.cell.2022.10.003 (2022).

79 Milacic, M. et al. The Reactome Pathway Knowledgebase 2024. Nucleic Acids Res 52, D672-D678, doi:10.1093/nar/gkad1025 (2024).

84 Huttlin, E. L. et al. The BioPlex Network: A Systematic Exploration of the Human Interactome. Cell 162, 425-440, doi:10.1016/j.cell.2015.06.043 (2015).

92 Pacholczyk, T., Blakely, R. D. & Amara, S. G. Expression cloning of a cocaine- and antidepressant-sensitive human noradrenaline transporter. Nature 350, 350-354, doi:10.1038/350350a0 (1991).

93 Erickson, J. D., Schafer, M. K., Bonner, T. I., Eiden, L. E. & Weihe, E. Distinct pharmacological properties and distribution in neurons and endocrine cells of two isoforms of the human vesicular monoamine transporter. Proc Natl Acad Sci U S A 93, 5166-5171, doi:10.1073/pnas.93.10.5166 (1996).

94 Arriza, J. L. et al. Functional comparisons of three glutamate transporter subtypes cloned from human motor cortex. J Neurosci 14, 5559-5569, doi:10.1523/JNEUROSCI.14-09-05559.1994 (1994).

95 Fairman, W. A., Vandenberg, R. J., Arriza, J. L., Kavanaugh, M. P. & Amara, S. G. An excitatory amino-acid transporter with properties of a ligand-gated chloride channel. Nature 375, 599-603, doi:10.1038/375599a0 (1995).

96 Parker, J. L. et al. Molecular basis for redox control by the human cystine/glutamate antiporter system xc(). Nat Commun 12, 7147, doi:10.1038/s41467-021-27414-1 (2021).

97 Fiermonte, G. et al. Identification of the mitochondrial glutamate transporter. Bacterial expression, reconstitution, functional characterization, and tissue distribution of two human isoforms. J Biol Chem 277, 19289-19294, doi:10.1074/jbc.M201572200 (2002).

98 Hellsten, S. V., Hagglund, M. G., Eriksson, M. M. & Fredriksson, R. The neuronal and astrocytic protein SLC38A10 transports glutamine, glutamate, and aspartate, suggesting a role in neurotransmission. FEBS Open Bio 7, 730-746, doi:10.1002/2211-5463.12219 (2017).

99 Wang, H. et al. Human placental Na+-dependent multivitamin transporter. Cloning, functional expression, gene structure, and chromosomal localization. J Biol Chem 274, 14875-14883, doi:10.1074/jbc.274.21.14875 (1999).

100 Indiveri, C., Palmieri, F., Bisaccia, F. & Kramer, R. Kinetics of the reconstituted 2-oxoglutarate carrier from bovine heart mitochondria. Biochim Biophys Acta 890, 310-318, doi:10.1016/0005-2728(87)90158-7 (1987).

101 Roberts, D. J. & Miyamoto, S. Hexokinase II integrates energy metabolism and cellular protection: Akting on mitochondria and TORCing to autophagy. Cell Death Differ 22, 364, doi:10.1038/cdd.2014.208 (2015).

102 Hackett, S. R. et al. Systems-level analysis of mechanisms regulating yeast metabolic flux. Science 354, doi:10.1126/science.aaf2786 (2016).

103 Britton, S., Alber, M. & Cannon, W. R. Enzyme activities predicted by metabolite concentrations and solvent capacity in the cell. J R Soc Interface 17, 20200656, doi:10.1098/rsif.2020.0656 (2020).

104 Hofmeyr, J. S. & Cornish-Bowden, A. Regulating the cellular economy of supply and demand. FEBS Lett 476, 47-51, doi:10.1016/s0014-5793(00)01668-9 (2000).

105 Tanner, L. B. et al. Four Key Steps Control Glycolytic Flux in Mammalian Cells. Cell Syst 7, 49-62 e48, doi:10.1016/j.cels.2018.06.003 (2018).

106 Cooney, G. J., Taegtmeyer, H. & Newsholme, E. A. Tricarboxylic acid cycle flux and enzyme activities in the isolated working rat heart. Biochem J 200, 701-703, doi:10.1042/bj2000701 (1981).

107 Butch, C. et al. Production of tartrates by cyanide-mediated dimerization of glyoxylate: a potential abiotic pathway to the citric acid cycle. J Am Chem Soc 135, 13440-13445, doi:10.1021/ja405103r (2013).
