# Supplementary material for: Covariation MS uncovers a protein that controls cysteine catabolism
Source: Nature. 2025 Sep 17;647(8088):268–76. doi: 10.1038/s41586-025-09535-5 (PMC12589099; doi:10.1038/s41586-025-09535-5)
Supplement: Supplementary file 2 — Reporting Summary [file 41586_2025_9535_MOESM2_ESM.pdf]

Corresponding author(s): Haopeng Xiao; Edward T. Chouchani

Last updated by author(s): Aug 2, 2025

Reporting Summary

Nature Portfolio wishes to improve the reproducibility of the work that we publish. This form provides structure for consistency and transparency in reporting. For further information on Nature Portfolio policies, see our Editorial Policies and the Editorial Policy Checklist.

Statistics

For all statistical analyses, confirm that the following items are present in the figure legend, table legend, main text, or Methods section.

n/a	Confirmed	
☐	☒	The exact sample size (*n*) for each experimental group/condition, given as a discrete number and unit of measurement
☐	☒	A statement on whether measurements were taken from distinct samples or whether the same sample was measured repeatedly
☐	☒	The statistical test(s) used AND whether they are one- or two-sided<br>*Only common tests should be described solely by name; describe more complex techniques in the Methods section.*
☐	☒	A description of all covariates tested
☐	☒	A description of any assumptions or corrections, such as tests of normality and adjustment for multiple comparisons
☐	☒	A full description of the statistical parameters including central tendency (e.g. means) or other basic estimates (e.g. regression coefficient) AND variation (e.g. standard deviation) or associated estimates of uncertainty (e.g. confidence intervals)
☐	☒	For null hypothesis testing, the test statistic (e.g. *F*, *t*, *r*) with confidence intervals, effect sizes, degrees of freedom and *P* value noted<br>*Give P values as exact values whenever suitable.*
☒	☐	For Bayesian analysis, information on the choice of priors and Markov chain Monte Carlo settings
☒	☐	For hierarchical and complex designs, identification of the appropriate level for tests and full reporting of outcomes
☐	☒	Estimates of effect sizes (e.g. Cohen's *d*, Pearson's *r*), indicating how they were calculated

Our web collection on statistics for biologists contains articles on many of the points above.

Software and code

Policy information about availability of computer code

Data collection	Chromeleon version 7.3.1, Xcalibur version 4.7.69.37
Data analysis	ColabFold V1.5, Alphafold-Multimer V2, R 4.2.0, cytoscape 3.9.1, Microsoft excel 16.98, Prism10, Masspike (Gygi lab)

For manuscripts utilizing custom algorithms or software that are central to the research but not yet described in published literature, software must be made available to editors and reviewers. We strongly encourage code deposition in a community repository (e.g. GitHub). See the Nature Portfolio guidelines for submitting code & software for further information.

Data

Policy information about availability of data

All manuscripts must include a data availability statement. This statement should provide the following information, where applicable:

- Accession codes, unique identifiers, or web links for publicly available datasets
- A description of any restrictions on data availability
- For clinical datasets or third party data, please ensure that the statement adheres to our policy

The mass spectrometry proteomics data have been deposited to the ProteomeXchange Consortium via the PRIDE partner repository with the dataset identifier PXD065355. Metabolomics data have been deposited to MassIVE under accession number MSV000098306. Other databases used in this work include UniProt (https://www.uniprot.org), Rhea (https://www.rhea-db.org), Reactome (https://reactome.org), TCDB (https://www.tcdb.org), and BioPlex (https://bioplex.hms.harvard.edu).

Research involving human participants, their data, or biological material

Policy information about studies with human participants or human data. See also policy information about sex, gender (identity/presentation), and sexual orientation and race, ethnicity and racism.

Reporting on sex and gender	N/A
Reporting on race, ethnicity, or other socially relevant groupings	N/A
Population characteristics	N/A
Recruitment	N/A
Ethics oversight	N/A

Note that full information on the approval of the study protocol must also be provided in the manuscript.

Field-specific reporting

Please select the one below that is the best fit for your research. If you are not sure, read the appropriate sections before making your selection.

☒ Life sciences ☐ Behavioural & social sciences ☐ Ecological, evolutionary & environmental sciences

For a reference copy of the document with all sections, see nature.com/documents/nr-reporting-summary-flat.pdf

Life sciences study design

All studies must disclose on these points even when the disclosure is negative.

Sample size	Sample sizes were determined based on previous experiments using similar methodologies to account for relevant biological and technical variability, then estimating statistical power based on methods described in PMID: 36334589
Data exclusions	One mouse in the control cohort of the in vivo LRRC58 experiment was excluded due to abnormal baseline LRRC58 transcript levels. Inclusion criteria were pre-established so that subjects in the control or LRRC58-AAV-KD cohort with baseline DFAC transcript levels failing Grubbs' test will be removed
Replication	All experimental findings were reproduced as biological replicates as the value stated in the figure legend, unless otherwise indicated. All additional replication attempts were successful
Randomization	For MS, samples were processed in random order, and sample allocation was randomized. For cell experiments, cells were grouped by treatments of genetic manipulations, and all cells were randomly seeded from the same seeding plate. For other animal experiments, all mice were housed under identical conditions, all experiments were comparing age- and sex-matched littermates. Mice were randomly selected into groups, based on genetic manipulation or treatments.
Blinding	Investigators were not blinded due to the nature of the experimental procedures, which required the researcher to be aware of group assignments for proper treatment administration and data collection.

Reporting for specific materials, systems and methods

We require information from authors about some types of materials, experimental systems and methods used in many studies. Here, indicate whether each material, system or method listed is relevant to your study. If you are not sure if a list item applies to your research, read the appropriate section before selecting a response.

Materials & experimental systems

n/a	Involved in the study	
☐	☒	Antibodies
☐	☒	Eukaryotic cell lines
☒	☐	Palaeontology and archaeology
☐	☒	Animals and other organisms
☒	☐	Clinical data
☒	☐	Dual use research of concern
☒	☐	Plants

Methods

n/a	Involved in the study	
☒	☐	ChIP-seq
☐	☒	Flow cytometry
☒	☐	MRI-based neuroimaging

Antibodies

Antibodies used	1. β-actin (CST, 3700) - 1:1000 dilution<br>2. Monoclonal ANTI-FLAG® M2 (Sigma, F3165) – 1:1000 dilution<br>3. Vinculin (CST, 4650) 1:1000 dilution<br>4. CDO1 polyclonal (ProteinTech, 12589-1-AP) 1:1000 dilution<br>5. CUL5 (Bethyl Laboratories, A302-173A) 1:1000 dilution<br>6. TCEB2/Elongin-B Polyclonal (ProteinTech, 10779-1-AP) 1:500 dilution<br>7. TCEB1/ELONGIN-C Polyclonal (ProteinTech, 12450-1-AP)<br>8. anti-rabbit HRP (Promega, W401B)- 1:10000 dilution<br>9. anti-mouse HRP (Promega, W402B) - 1:10000 dilution<br>10. CDO1 (1:1000 dilution anti-CDO1, Life Technologies 12589-1-AP).<br>11. Anti-rabbit secondary antibody (1:4000 dilution Anti-Rabbit IgG, LI-COR, Cat #92632211).
Validation	Above antibodies have all been validated by manufacturer. Sources of validation are provided below<br>1. β-actin (CST, 3700) - 1:1000 dilution<br>https://www.cellsignal.com/products/primary-antibodies/b-actin-8h10d10-mouse-mab/3700?srsltid=AfmBOooVCl4FJVvtds8yhD2p4Lfjo_YFTBEx3tdx0IRAbtDJ653Xkmub<br>2. Monoclonal ANTI-FLAG® M2 (Sigma, F3165) – 1:1000 dilution<br>https://www.sigmaaldrich.com/US/en/product/sigma/f3165?srsltid=AfmBOorl7SSwmC3cD-s0q1Z_4LhylXpQBG6S6o7yWOAtofy503ebI1o3<br>3. Vinculin (CST, 4650) 1:1000 dilution<br>https://www.cellsignal.com/products/primary-antibodies/vinculin-antibody/4650?srsltid=AfmBOoqEenoVhynno0fz4lbHuaNOVN34NrIM_QUmgmrVl2MxSHHdMkq9<br>4. CDO1 polyclonal (ProteinTech, 12589-1-AP) 1:1000 dilution<br>https://www.ptglab.com/products/CDO1-Antibody-12589-1-AP.htm?srsltid=AfmBOopWlitmbZPH58ZjqWybm3PezonT1wtVAiCrkk76x_N1a1qd7Z_8<br>5. CUL5 (Bethyl Laboratories, A302-173A) 1:1000 dilution<br>https://www.fortislife.com/products/primary-antibodies/rabbit-anti-cul5-antibody/BETHYL-A302-173?selected=A302-173A<br>6. TCEB2/Elongin-B Polyclonal (ProteinTech, 10779-1-AP) 1:500 dilution<br>https://www.ptglab.com/products/TCEB2-Antibody-10779-1-AP.htm?srsltid=AfmBOooVaUS7UdlETjlhc7N74pIw8v4Zse5rkj2KrtJVT1lM4ZUdylyk<br>7. TCEB1/ELONGIN-C Polyclonal (ProteinTech, 12450-1-AP)<br>https://www.ptglab.com/products/TCEB1-Antibody-12450-1-AP.htm?srsltid=AfmBOoo0MI_2T-AyogVv8Oqpe2vK-lDQh3JYcaDRT-j_nQ6H27Y3_A1p<br>8. anti-rabbit HRP (Promega, W401B)- 1:10000 dilution<br>https://www.promega.com/products/protein-detection/primary-and-secondary-antibodies/anti-rabbit-igg-h-and-l-hrp-conjugate/?catNum=W4011<br>9. anti-mouse HRP (Promega, W402B) - 1:10000 dilution<br>https://www.promega.com/products/protein-detection/primary-and-secondary-antibodies/anti_mouse-igg-h-and-l-hrp-conjugate/?catNum=W4021<br>10. CDO1 (1:1000 dilution anti-CDO1, Life Technologies 12589-1-AP).<br>https://www.thermofisher.com/antibody/product/CDO1-Antibody-Polyclonal/12589-1-AP<br>11. Anti-rabbit secondary antibody (1:4000 dilution Anti-Rabbit IgG, LI-COR, Cat #92632211).<br>https://www.licorbio.com/support/contents/reagents/irdye-secondary-antibodies/800cw/goat-anti-rabbit-igg.html

Eukaryotic cell lines

Policy information about cell lines and Sex and Gender in Research

Cell line source(s)	ATCC- Hep G2; primary hepatocytes prepared from C56BL/6J mice (mice purchased from Jackson lab); primary brown adipocytes prepared from C56BL/6J mice (mice purchased from Jackson lab);
Authentication	ATCC cells were validated by ATCC using STR profiling: https://www.atcc.org/en/services/cell-authentication
Mycoplasma contamination	Cells tested negative for mycoplasma.
Commonly misidentified lines (See ICLAC register)	None used

Animals and other research organisms

Policy information about studies involving animals; ARRIVE guidelines recommended for reporting animal research, and Sex and Gender in Research

Laboratory animals	C57BL/6J mice (8 - 12 weeks) were obtained from Jackson Laboratories were used. Female diversity outbred (DO) mice (24 weeks, n=110; 18 months, n=10; 22 months, n=29; 28 months, n=14) were also obtained from Jackson lab. Mice were housed in a temperature controlled room (23°C) on a 12h light-dark cycle
Wild animals	None used

Reporting on sex | Diversity Outbred mice were all females due to availability; C57BL/6J mice were all males

Field-collected samples | Non used

Ethics oversight | All animal-related experiments were approved by Institutional Animal Care and Use Committee of the Beth Israel Deaconess Medical Center

Note that full information on the approval of the study protocol must also be provided in the manuscript.

Plants

Seed stocks | N/A

Novel plant genotypes | N/A

Authentication | N/A

Flow Cytometry

Plots

Confirm that:

☒ The axis labels state the marker and fluorochrome used (e.g. CD4-FITC).

☒ The axis scales are clearly visible. Include numbers along axes only for bottom left plot of group (a 'group' is an analysis of identical markers).

☒ All plots are contour plots with outliers or pseudocolor plots.

☒ A numerical value for number of cells or percentage (with statistics) is provided.

Methodology

Sample preparation | HepG2 cells were purchased from ATCC and trasfected with the CDO1 stability reported as detailed in the method section. Cells were cultured on 96 well plates and treated with the appropriate compound/media. They were then washed with PBS, trypsinized, and resuspended in PBS with 10% FCS and DAPI for analysis on an HTS plate reader

Instrument | BD biosciences, LSR Fortessa with HTS

Software | FlowJo v10

Cell population abundance | mCherry positive cells were >80% of live cellular events

Gating strategy | We gated on FSC/SSC, then DAPI negative cells (as determined by unstained control) then mCherry+ cells (as determined by uninfected control). We evaluated the ratio of the GFP MFI to the mCherry MFI in this population

☒ Tick this box to confirm that a figure exemplifying the gating strategy is provided in the Supplementary Information.
