# Supplementary material for: Covariation MS uncovers a protein that controls cysteine catabolism
Source: Nature. 2025 Sep 17;647(8088):268–76. doi: 10.1038/s41586-025-09535-5 (PMC12589099; doi:10.1038/s41586-025-09535-5)

Figure 3g

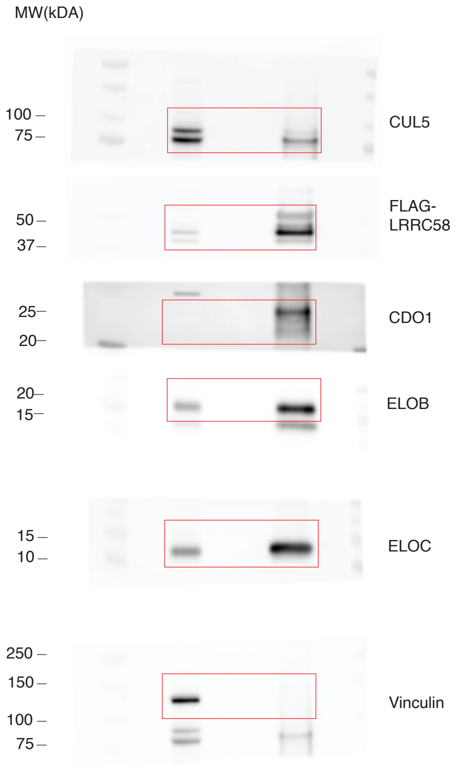

Extended Figure 7c

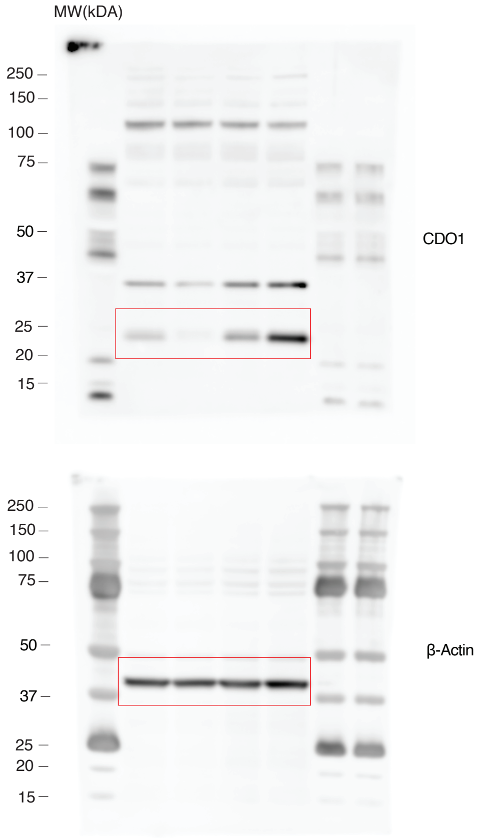

Extended Figure 7e

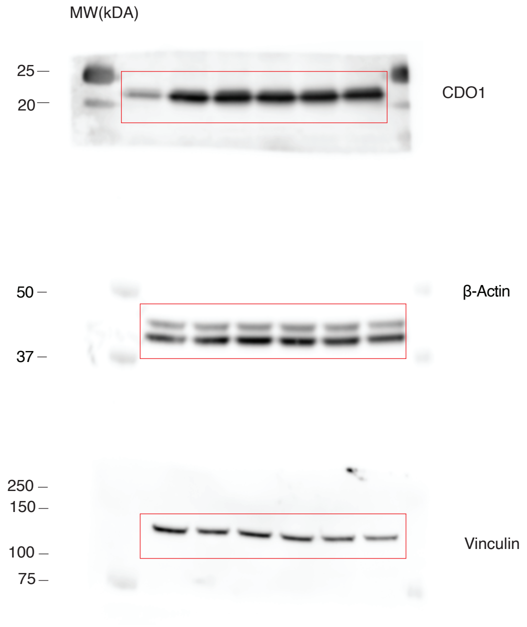

Extended Figure 7f

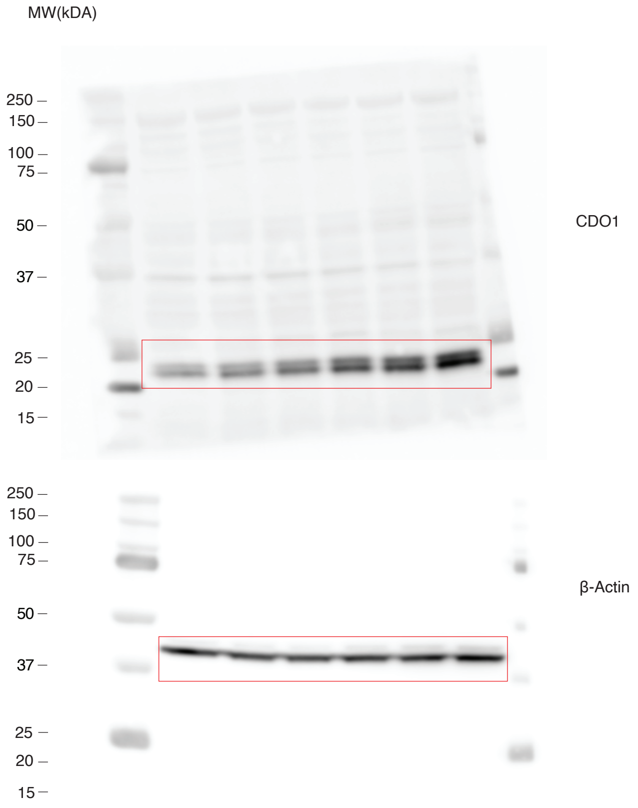

Extended Figure 7g

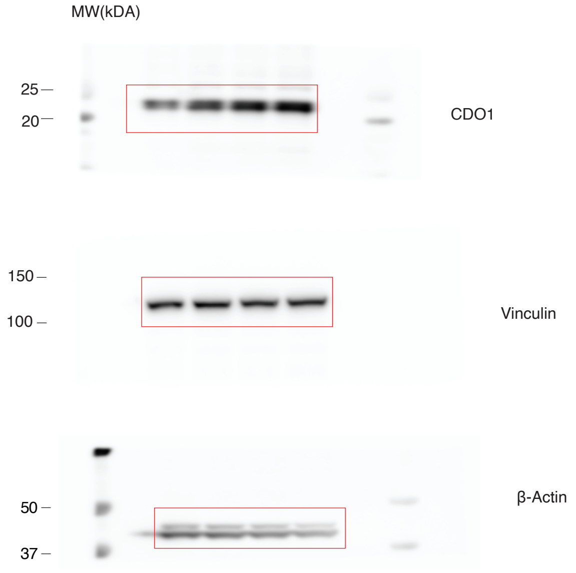

Extended Figure 7h

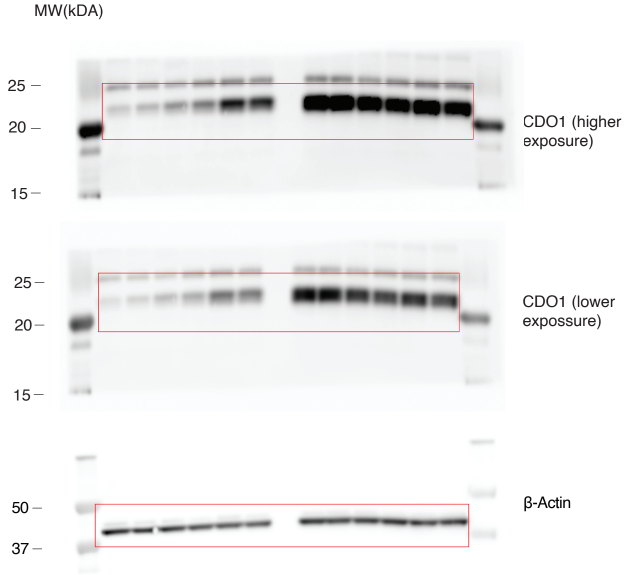

Extended Figure 7j

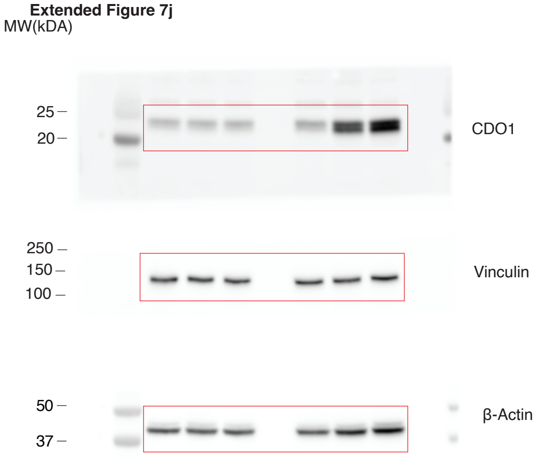

Extended Figure 7i

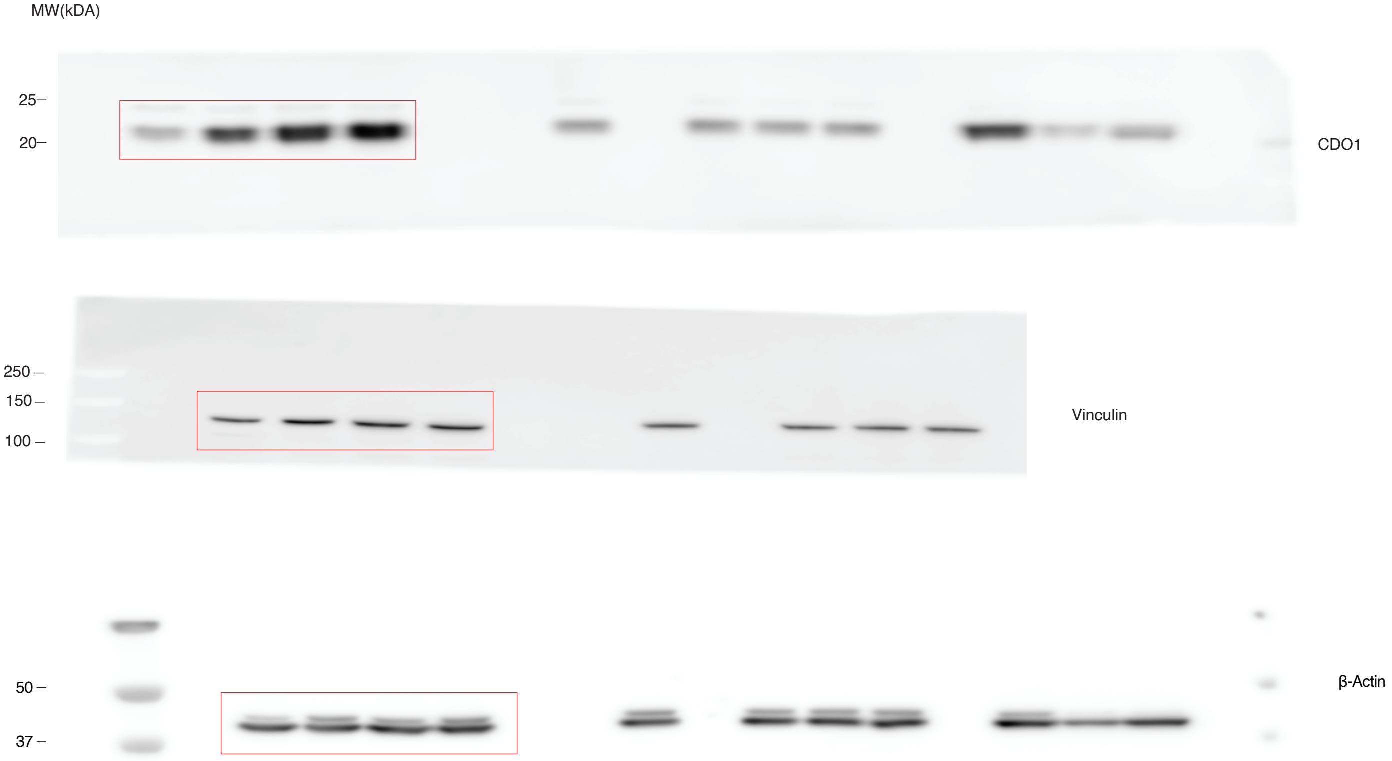

Extended Figure 7k

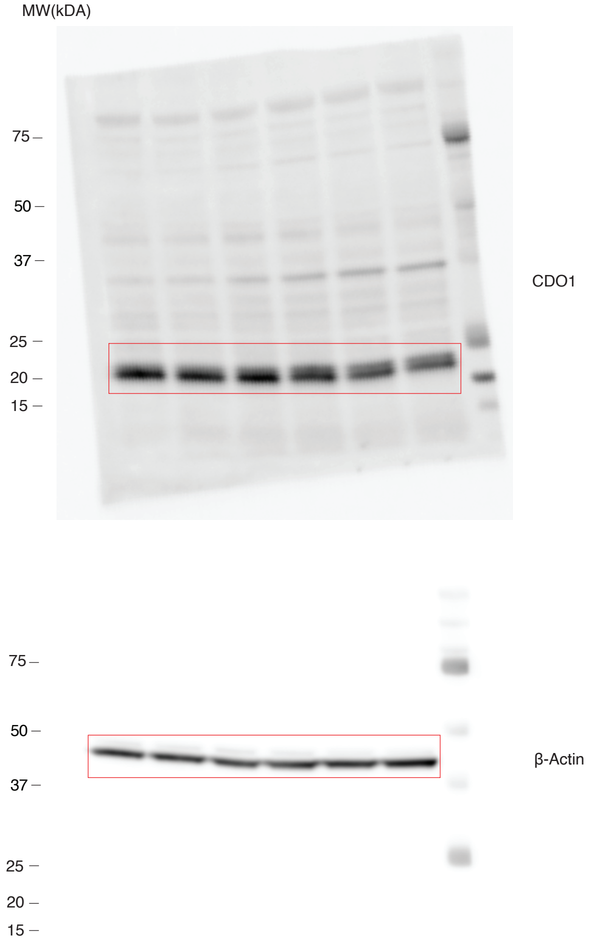

Extended Figure 7l

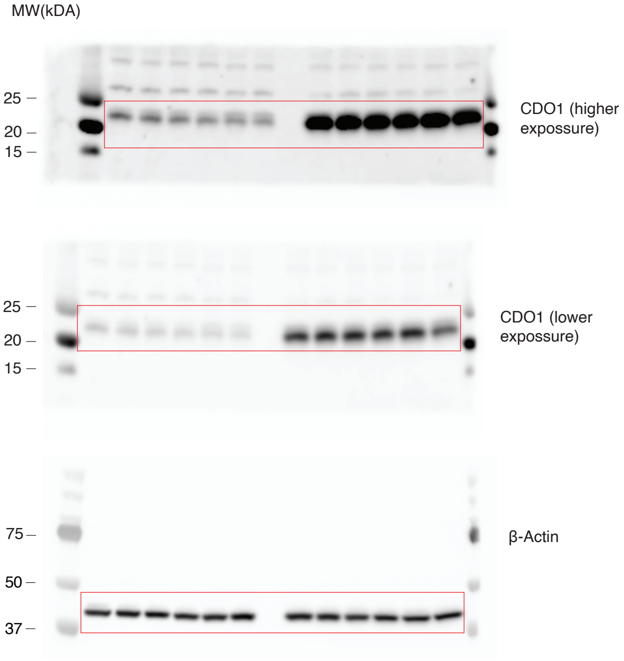

Extended Figure 8l

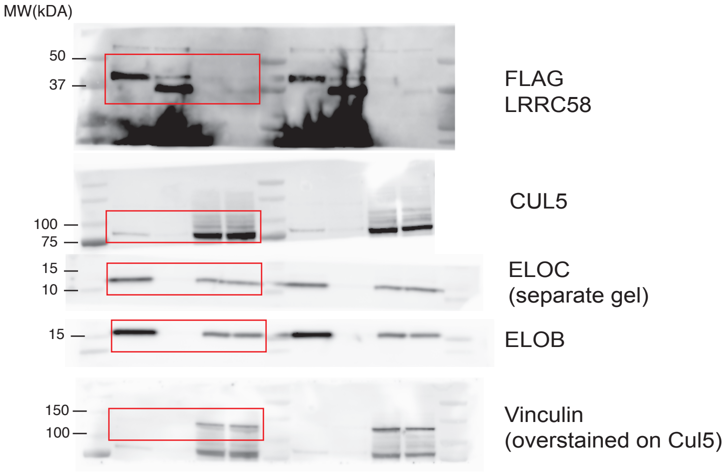

Supplement: Supplementary file 3 — Uncropped western blots. [file 41586_2025_9535_MOESM3_ESM.pdf]
