# Supplementary figures and images for: Covariation MS uncovers a protein that controls cysteine catabolism
Source: Nature. 2025 Sep 17;647(8088):268–76. doi: 10.1038/s41586-025-09535-5 (PMC12589099; doi:10.1038/s41586-025-09535-5)

Supplementary Figure 1:

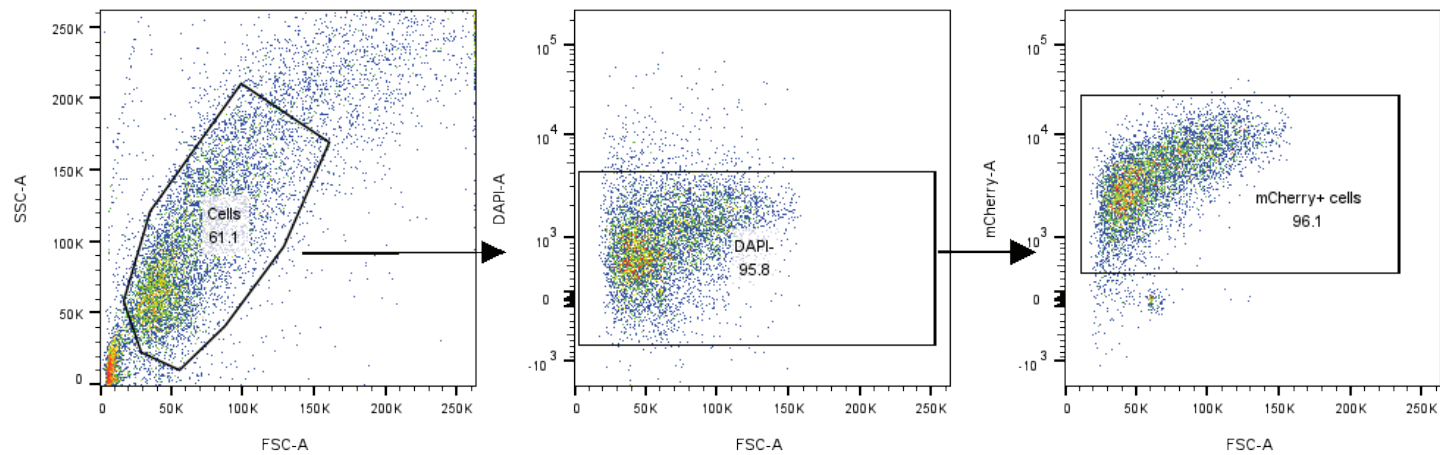

Supplement: Supplementary file 4 — Example flow cytometry gating. [file 41586_2025_9535_MOESM4_ESM.pdf]
