# Supplementary material for: Covariation MS uncovers a protein that controls cysteine catabolism
Source: Nature. 2025 Sep 17;647(8088):268–76. doi: 10.1038/s41586-025-09535-5 (PMC12589099; doi:10.1038/s41586-025-09535-5)
Supplement: Supplementary file 5 — Supplementary Tables 1–10. [file 41586_2025_9535_MOESM5_ESM.zip › 2024-04-07889D-s5/Supplementary Table legends.docx]

Supplementary Table 1- Quantification of 11,867 proteins and 285 metabolites from brown adipose tissue (BAT) and liver of DO mice

Supplementary Table 2- 482,043 Co-operative protein-metabolite edges

Supplementary Table 3- MPCA recapitulation of established biochemical reactions, pathways, and metabolite transporters

Supplementary Table 4- Accessory members of metabolic pathways identified using MPCA

Supplementary Table 5- Protein predictors of metabolite abundance based on LASSO modeling

Supplementary Table 6- Metabolomics of knockdown and overexpression of LRRC58 in primary brown adipocytes, primary hepatocytes, and Hep G2 cells

Supplementary Table 7- Proteomics of knockdown and overexpression of LRRC58 in primary brown adipocytes, primary hepatocytes, and Hep G2 cells

Supplementary Table 8- MS quantification of LRRC58 protein abundance in western blotting experiments

Supplementary Table 9- Proteomics and metabolomics of LRRC58 knockdown in mouse liver

Supplementary Table 10- CDO1 and LRRC58 single nucleotide polymorphisms (SNPs)
